# Women's Education Level, Maternal Health Facilities, Abortion Legislation and Maternal Deaths: A Natural Experiment in Chile from 1957 to 2007

**DOI:** 10.1371/journal.pone.0036613

**Published:** 2012-05-04

**Authors:** Elard Koch, John Thorp, Miguel Bravo, Sebastián Gatica, Camila X. Romero, Hernán Aguilera, Ivonne Ahlers

**Affiliations:** 1 Institute of Molecular Epidemiology (MELISA), Center of Embryonic Medicine and Maternal Health, Faculty of Medicine, Universidad Católica de la Santísima Concepción, Concepción, Chile; 2 Department of Primary Care and Family Medicine, Faculty of Medicine, University of Chile, Santiago, Chile; 3 Doctoral Program, Division of Epidemiology, School of Public Health, Faculty of Medicine, University of Chile, Santiago, Chile; 4 Department of Obstetrics and Gynecology, University of North Carolina-Chapel Hill, Chapel Hill, North Carolina, United States of America; The University of Adelaide, Australia

## Abstract

**Background:**

The aim of this study was to assess the main factors related to maternal mortality reduction in large time series available in Chile in context of the United Nations' Millennium Development Goals (MDGs).

**Methods:**

Time series of maternal mortality ratio (MMR) from official data (National Institute of Statistics, 1957–2007) along with parallel time series of education years, income per capita, fertility rate (TFR), birth order, clean water, sanitary sewer, and delivery by skilled attendants were analysed using autoregressive models (ARIMA). Historical changes on the mortality trend including the effect of different educational and maternal health policies implemented in 1965, and legislation that prohibited abortion in 1989 were assessed utilizing segmented regression techniques.

**Results:**

During the 50-year study period, the MMR decreased from 293.7 to 18.2/100,000 live births, a decrease of 93.8%. Women's education level modulated the effects of TFR, birth order, delivery by skilled attendants, clean water, and sanitary sewer access. In the fully adjusted model, for every additional year of maternal education there was a corresponding decrease in the MMR of 29.3/100,000 live births. A rapid phase of decline between 1965 and 1981 (−13.29/100,000 live births each year) and a slow phase between 1981 and 2007 (−1.59/100,000 live births each year) were identified. After abortion was prohibited, the MMR decreased from 41.3 to 12.7 per 100,000 live births (−69.2%). The slope of the MMR did not appear to be altered by the change in abortion law.

**Conclusion:**

Increasing education level appears to favourably impact the downward trend in the MMR, modulating other key factors such as access and utilization of maternal health facilities, changes in women's reproductive behaviour and improvements of the sanitary system. Consequently, different MDGs can act synergistically to improve maternal health. The reduction in the MMR is not related to the legal status of abortion.

## Introduction

The fifth Millennium Development Goal (MDG) put forward by the United Nations (MDG-5) proposes to reduce the world's maternal mortality ratio by 75%, by 2015 [1]. Many pregnancy-related deaths are preventable, and maternal mortality remains high in Latin America [2]. Nevertheless, according to a recent independent study of 181 countries by Hogan *et* *al.* [3], and contrary to previous reports that showed very little decrease in the maternal mortality ratio (MMR, the number of maternal deaths related to childbearing divided by the number of live births) over decades [4]–[6], the global MMR declined from 422 to 251 per 100,000 live births between 1980 and 2008. In particular, low-income Latin American developing countries, such as El Salvador, Guatemala, Nicaragua, Ecuador, and Bolivia, have made substantial progress in reducing the MMR [3].

Factors such as fertility rate [3], [5]–[9] (a proxy for reproductive behaviour), per capita income [10] (an indicator of material resources in adult life), educational attainment of the female population [3], [11]–[15] (an indicator of early life experiences, acquired knowledge and skills [16]), access to adequate maternal health facilities and personnel (*e.g.* skilled attendants) are all thought to be important determinants of maternal health [17]–[21]. Additionally, it has been suggested that abortion prohibition may contribute to high maternal mortality rates [22]–[26]. Finally, although the influence of other development process indicators such as clean water supply and sanitary sewer access on maternal mortality is virtually unknown, these factors likely influence population health by decreasing epidemics and mortality from diarrhoeal infectious diseases [27], [28].

Chile offers an opportunity to investigate the influence of these determinants on maternal mortality trends. Not only are large time series of vital and socioeconomic data available for this country that are of similar quality to those of developed countries [2], [29], but legislation prohibiting therapeutic abortion was passed in 1989. As a result, data from Chile provide a rare and unique natural experiment to evaluate the influence of population factors, the legal status of abortion and other historical policies on maternal mortality trends since data are available before and after interventions were implemented.

Interestingly, research has consistently observed an inverse correlation between women's education level and maternal mortality in the developing world [11], [12], [14], [15], [30]. Recent Chilean prospective studies have corroborated the finding that educational attainment is a strong independent predictor of all-cause mortality having simultaneously a modulating effect on other factors [16], [31]. Although it has been suggested that increasing women's education level contributes to the modulation of other variables known to influence maternal health such as the reproductive behaviour (*e.g.* fertility rate, birth order, delayed marriage and motherhood, family size, contraceptive use, etc.) and access to maternal health facilities (*e.g.* access to prenatal and postnatal care, and delivery by skilled attendants) [3], [7], [11]–[15], [30], this effect on the MMR decline has yet to be demonstrated mathematically; however, this may be severely limited by the paucity of large and continuous parallel time series in developing countries [7], [20], [32], [33].

The primary objective of this study is to assess the main factors related to maternal mortality reduction in Chile over the last fifty years, including the implementation of historical policies. A complementary objective is to test the modifying effect of women's education level on other variables identified to influence the mortality trend. Accordingly, we use a parallel time series design combining segmented regression techniques with a pathway modelling approach.

## Methods

### Ethics statement

The ethical aspects of this study were reviewed and approved by the institutional review board of the Faculty of Medicine of the University of Chile. Since this work was based on historical mortality statistics and did not directly involve any human subjects, this institutional review board explicitly waived the need for informed consent.

### Maternal deaths and live births

We systematically searched the Chilean National Institutes of Statistics (INE) for official government data on maternal deaths and live births from 1957 to the present. Vital registration in Chile is virtually complete, and the quality of the data meets the standards of developed countries [29], [34], [35]. In our analysis of official vital statistic yearbooks that have been published continually from 1957 by the INE, we identified four well-defined periods of registry, during which specific international classification of diseases (ICD) were utilised in Chile. During the first period from 1958 to 1967, causes of maternal mortality were classified according to ICD-7 (7^th^ version). In 1957 the ICD-6 (6^th^ version) was used but obstetric causes were directly homologated with ICD-7. In the second period from 1968 to 1979, maternal deaths were classified using ICD-8 (8^th^ version). From 1980 to 1996, the ICD-9 (9^th^ version) was used, and from 1997 to the present, ICD-10 (10^th^ version) has been used. The detailed description to classify death causes is presented in Appendix S1 and Tables S1, S2, S3, S4 and S5. Regarding live births, between 1957 and 1979 there was an increase in the delay of the inscription of births [36]. Therefore, in this study, the number of live births for every year was corrected using the method of delayed registration (Appendix S1).

### Independent variables

Women's education level was assessed with the construction of parallel time series using the average number of schooling years (Appendix S1). First, we used the series published by the Central Bank of Chile [37] and by the Economic History and Cliometrics Laboratory [38]. Second, we used estimates from the Social Characterization Survey conducted by the Chilean Ministry of Planning to calculate the average number of schooling years for economically active female population. Finally, we used the percentage of women with nine or more schooling years at the time of delivery, published by the INE in the annual registry of live births from 1957 to 2007. To obtain a single set of 51 points representing the average number of schooling years, we used a multiple regression method [39], including all of the variables mentioned above. Other independent predictors that were analysed in continuous and parallel times series were income per capita (Gross Domestic Income [GDI] in U.S. dollars), percent of population with clean water supply, percent of population with sanitary sewer access, total fertility rate (TFR), birth order (percent of primiparous women giving birth and percent of primiparous women >29 years giving birth from the total live births each year), and percentage of women who were delivered by skilled attendants (deliveries by medical doctors and/or professional midwifes in hospitals or maternities). The conceptual definitions and the procedures for collecting all parallel time series data are detailed in Appendix S1.

### Historical interventions

In our analysis we considered three historical interventions and policies that may have influenced women's health and consequently the MMR (a full description is provided in Appendix S1). First, in 1965, laws were passed implementing free and mandatory education to a minimum of eight years [40]. Second, between 1964 and 1967, an extensive prenatal primary care program with a family planning component was implemented [41]. Finally, in 1989, a legislation prohibiting therapeutic abortion was passed, completing all historical interventions considered.

### Statistical analyses

The MMR per 100,000 live births was directly calculated from the official registry of maternal deaths and live births. Additionally, because it was not possible to completely align all ICD codes, we computed the relative importance of various causes of maternal mortality, grouping similar causes of maternal deaths together while attempting to work within the context of the original codification (Table S1, Appendix S1). In particular, ICD-9 and ICD-10 included separate codes for several causes that were not included in the ICD-7 or ICD-8. Therefore, the oldest ICD was used as a reference to construct the various mortality groups and to calculate the relative importance in four five-year periods: 1958–1962, 1971–1975, 1985–1989 and 2003–2007. In exploratory statistical analyses using a spline smoothing procedure for the number of maternal deaths, these four five-year periods were parsimoniously representative of the four ICD codes used in the time series. Additional analysis to identify changes in the trend for the percent of mortality causes included continuous five-year intervals. This procedure allows minimizing possible estimation errors of any specific cause associated to time-dependent outliers in the time series.

Correlations of every variable with the MMR were explored through the *R^2^* coefficient. To assess the impact of independent variables on the maternal mortality trend, we employed an autoregressive integrated moving average (ARIMA) model. Autocorrelation functions for each time series showed that differentiating at order-one was sufficient for control by the autoregressive (AR) component. To assess the effect of historical interventions, a segmented regression technique [42] that sequentially incorporated join points and their corresponding segments into multiple ARIMA models was used. In addition, visual inspection of the scatterplot was used to identify other possible join points. Finally, the impact of each independent variable on the linear terms of the MMR (changes in the *β*-coefficients with two tailed p-values) was evaluated in sequential ARIMA models using a pathway modelling approach, under the hypothesis that the mean number of years of female education modulates the slopes of all other predictors as an antecedent variable.

## Results

### Maternal mortality trend

**Figure 1:**
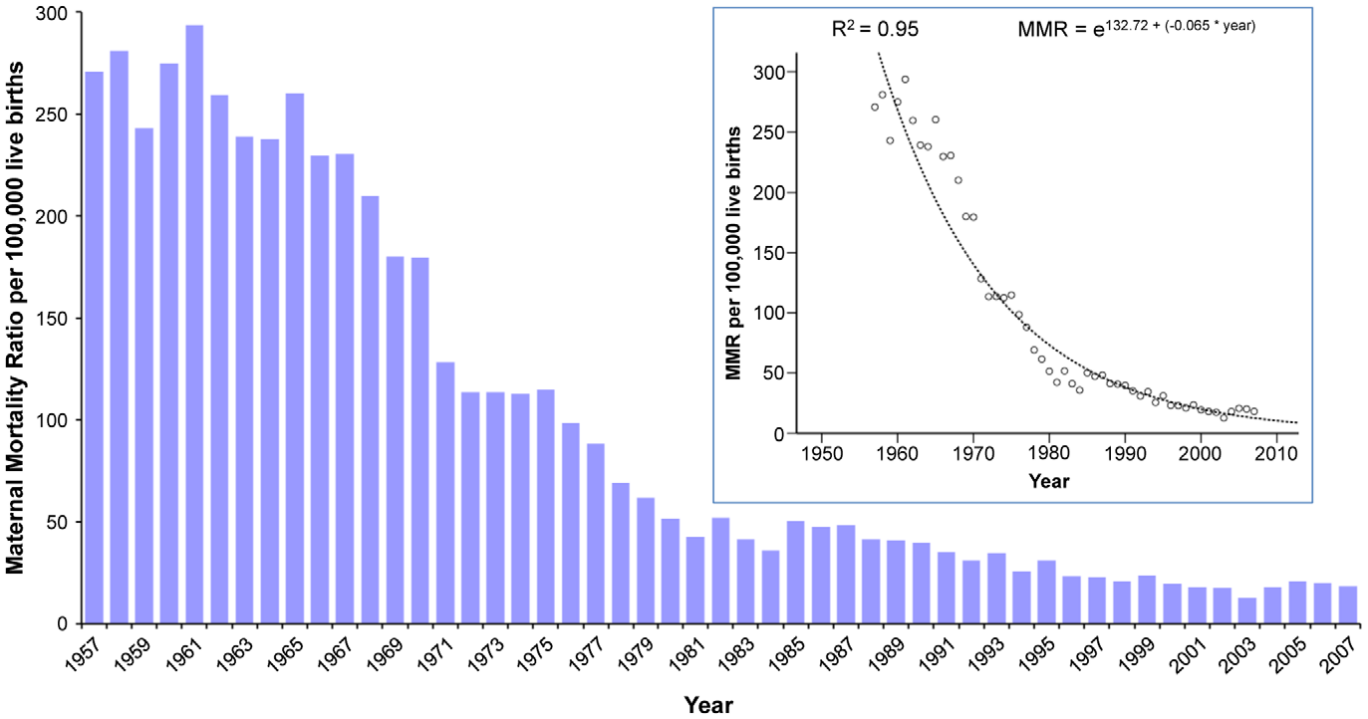
Trend for maternal mortality ratio, Chile 1957–2007. The secondary graphic shows the best adjustment of total trend for Maternal Mortality Ratio (MMR) over time.

During the period from 1957 to 2007 there were 14,413 maternal deaths and 13,799,330 live births in Chile, corresponding to a MMR of 102.3 per 100,000 live births. The MMR was 270.7 per 100,000 in 1957, decreasing to 18.2 per 100,000 live births in 2007 (Figure 1). This represented a total reduction of 93.7%. The highest MMR was observed in 1961, with 293.7 per 100,000 live births, and the lowest in 2003, with 12.7 per 100,000 live births. The best estimated curve for the total trend over time was exponential with a goodness-of-fit of 95.9%. The maternal mortality rate displayed similar trends (Table S6, Appendix S2). The highest mortality rate was observed in 1961, at 47.9 per 100,000 women of reproductive age (15–49 years), and the lowest was observed in 2003, reaching a rate of 0.72 per 100,000 women. The accumulated decrease for the total time period was −43.7 per 100,000 women (a reduction of 97.8%).

### Main causes of maternal death

**Figure 2:**
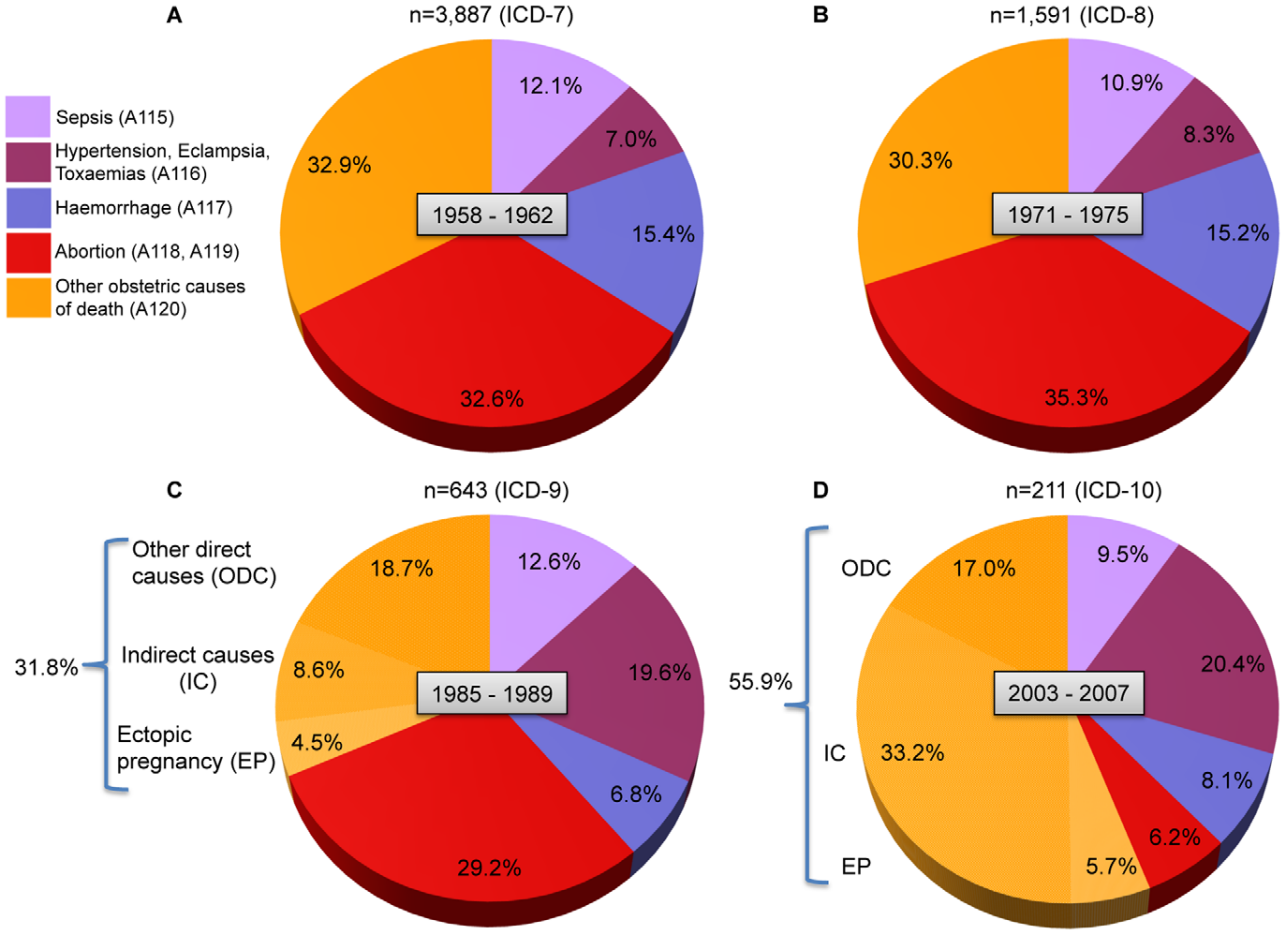
Relative importance of different causes of maternal death in four periods in Chile between 1957 and 2007. Pie chart A represents the period from 1958 to 1962; B period from 1971 to 1075; C period from 1985 to 1989; and D period from 2003 to 2007. Each period shows the five major causes of maternal mortality according to international codes of disease. Code homologation was carried out using ICD 7^th^ version (ICD-7) as reference. Causes of death present in ICD versions 8, 9 and 10 were grouped using ICD-7 codes A115 (Sepsis); A116 (Hypertension, Eclampsia and Toxaemias); A117 (Haemorrhage); A118 and A119 (Abortion); A120 (Other direct and indirect obstetric causes of death, including ectopic pregnancy, hydatidiform mole and other abnormal products of conceptions). Pie charts C and D include a more specific subgroup for death causes from ICD-9 and ICD-10.

The percentage of mortality due to abortion, sepsis and other direct and indirect causes of maternal death as defined by the International Classification of Diseases remained unchanged over the first three periods, suggesting that all of these causes declined proportionally between 1957 and 1989 (Figure 2). Abortion (whether spontaneous or induced but excluding ectopic pregnancy, hydatidiform mole, and other abnormal products of conception classified in other direct and indirect causes of mortality; Table S2, Appendix S1,) was the leading cause of maternal death in the first three periods (over 30%). In the third period (1985–1989), the percentages of maternal deaths due to hypertension, eclampsia and toxaemias increased and that for haemorrhage decreased. In the last period from 2003 to 2007, the leading causes of death were indirect, mainly represented by non-obstetric pre-existing chronic conditions. In additional analyses of continuous five-year intervals, maternal deaths by indirect causes increased in percent beyond the limit of 10% from the interval 1991–95 (Figure S1).

**Figure 3:**
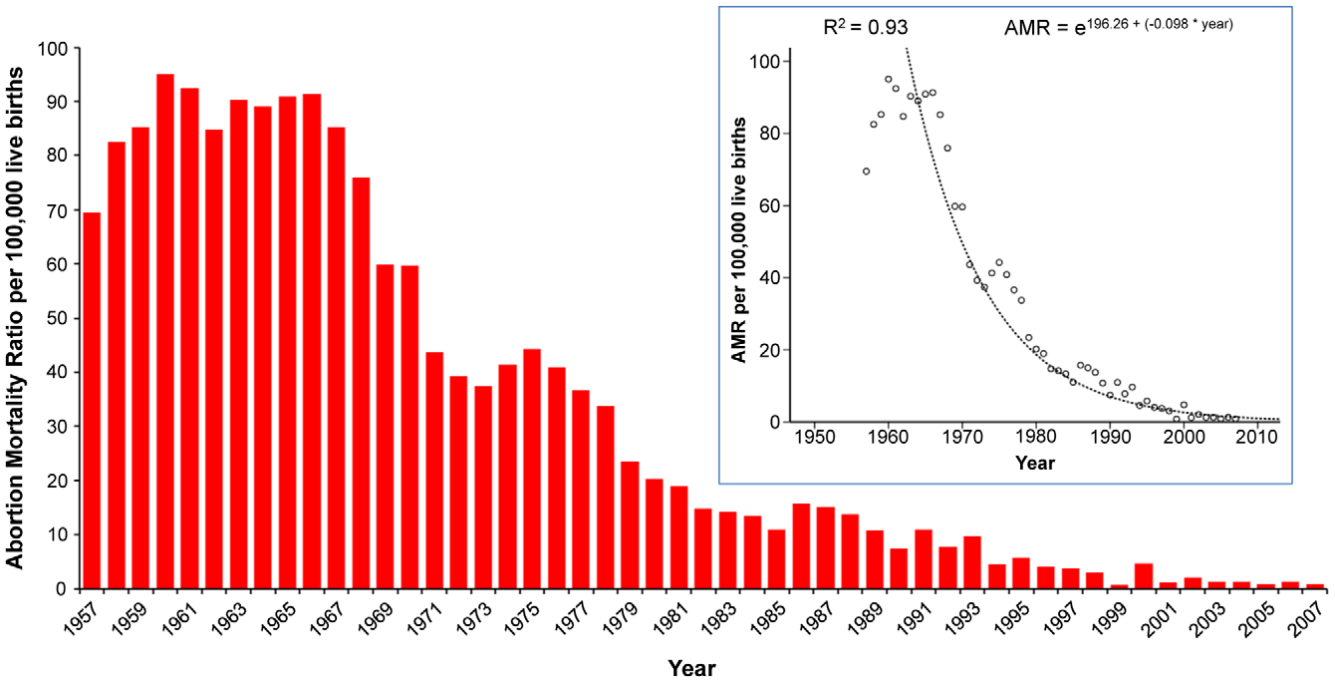
Trend for abortion mortality ratio (AMR), Chile 1957–2007. The highest AMR was observed in 1961, with 95.1 per 100,000 live births decreasing to 0.83 per 100,000 live births in 2007. This represented an accumulated reduction of 99.1%. The best estimated curve for the total trend over time was exponential with a goodness-of-fit of 93.5% (secondary chart). In 1989, the year of abortion prohibition, AMR was 10.78 per 100,000 live births. The accumulated decrease for the period between 1989 and 2007 was −9.95 per 100,000 live births (a reduction of 92.3% from 1989).

Figure 3 shows the complete trend for abortion mortality ratio per 100,000 live births. Although abortion mortality ratio (number of deaths due to abortion divided by the number of live births) had decreased continuously, the percent of deaths related to abortion declined progressively under the limit of 35% starting on interval 1981–85 (Figure S2). In absolute terms, two deaths from ectopic pregnancy (code O00) and two from unspecified abortion (code O06) were observed in 2007. The abortion mortality ratio was 0.83 per 100,000 live births, and the absolute risk of dying from abortion was 0.046 per 100,000 women at fertile age –one in two million women between 15 and 49 years old.

### Parallel time series

The complete time series analysed in the study are presented in Table 1. TFR showed a continuous decreasing trend from 1964 to 1979, from 4.6 to 2.4, respectively. Between the years 1980 and 1990, the trend stopped, showing a slight increase to 2.7. From 1991, the TFR decreased from 2.6 to 1.88 in 2007. The percentage of primiparous women increased from 22.8% in 1964 to 40% in 1978, remaining close to this value until 2003 and then rapidly increasing to 45% in 2007. The percentage of primiparous women giving birth greater than 29 years old remained between 2.7 and 3.3% between the years 1957 and 1984, respectively (median and mode equal to 3%) and progressively increased from 3.4 to 7.2% between 1985 and 2007. The women's education level had average values between 3.1 and 4.4 years of schooling between 1957 and 1964. From 1965, the average number of schooling years increased continuously from 4.6 to 12. In 1957, the proportion of deliveries by skilled attendants was 60.8% and increased continuously, reaching 99.1% in 1990. This trend increased, reaching 99.8% in 2007. GDI per capita (PPP) increased from US$4,031 to US$5,929 between 1957 and 1988. This value has continued to increase since 1989, reaching US$13,556 in 2007. Finally, clean water coverage and sanitary sewers have increased continuously, reaching 99.9% and 95.2%, respectively, in 2007.

**Table 1:** Parallel time series of the co-variables assessed in the study on maternal mortality in Chile from 1957 to 2007.

Year	TFR	Primiparous (%)	Primiparous >29 years (%)	Education, years†	Skilled attendants (%)	GDI per capita (US$)	Clean water (%)	Sanitary sewer (%)
1957	5.0	23.9	3.0	3.57	60.8	4,250	25.5	12.1
1958	5.0	23.1	3.0	3.43	62.4	4,376	29.2	13.9
1959	4.8	23.0	3.2	4.31	59.8	4,031	32.8	15.6
1960	5.0	23.3	3.3	3.57	66.9	4,265	36.3	16,7
1961	5.1	22.9	3.0	3.14	69.7	4,354	39.7	17.9
1962	5.1	23.2	3.1	3.93	72.1	4,447	43.0	19.4
1963	5.0	22.7	2.7	4.40	72.3	4,613	44.8	21.3
1964	4.6	22.8	2.8	4.43	73.0	4,604	49.2	23.4
1965	4.6	24.2	2.8	3.91	74.3	4,533	53.5	25.4
1966	4.3	25.4	2.8	4.62	75.5	4,933	56.3	26.0
1967	3.8	27.1	2.9	4.60	77.3	4,989	59.1	26.8
1968	3.8	28.3	2.9	5.07	78.0	5,064	61.7	27.8
1969	3.3	29.9	3.1	5.76	80.0	5,149	64.1	29.5
1970	3.4	31.1	3.1	5.53	81.1	5,154	66.5	31.1
1971	3.3	31.8	3.2	6.95	83.6	5,518	67.2	33.0
1972	3.3	33.3	3.4	7.29	85.0	5,358	67.9	34.8
1973	3.6	34.5	3.3	7.29	85.1	4,975	68.6	36.5
1974	3.5	34.0	3.3	7.31	86.4	4,941	69.2	38.2
1975	3.2	35.4	3.3	7.26	87.4	4,233	77.4	43.5
1976	2.7	36.6	3.3	8.20	87.1	4,319	78.2	51.5
1977	2.7	38.4	3.4	8.44	89.0	4,678	82.6	55.9
1978	2.4	40.1	3.5	8.31	89.6	4,991	86.0	56.3
1979	2.4	40.4	3.4	8.49	90.4	5,330	90.1	62.4
1980	2.5	40.6	3.3	8.40	91.4	5,675	91.4	67.4
1981	2.6	40.2	3.3	8.43	92.2	5,929	91.5	68.2
1982	2.6	38.8	3.2	8.56	94.2	5,041	92.1	70.0
1983	2.6	38.4	3.3	8.67	95.2	4,822	92.7	70.6
1984	2.5	39.0	3.3	8.86	96.7	5,027	94.3	72.9
1985	2.5	39.8	3.4	9.04	97.4	5,047	95.2	75.1
1986	2.5	40.8	3.6	8.90	97.7	5,241	97.0	77.2
1987	2.5	41.0	3.7	8.95	98.1	5,484	97.2	78.8
1988	2.7	41.1	3.9	9.07	98.4	5,787	98.0	80.8
1989	2.7	41.0	4.0	9.18	98.8	6,293	98.2	81.5
1990	2.7	40.5	4.2	9.14	99.1	6,424	97.4	82.6
1991	2.6	39.8	4.4	9.17	99.2	6,807	95.3	86.2
1992	2.5	39.5	4.4	10.06	99.2	7,500	97.5	84.7
1993	2.5	39.9	4.6	10.12	99.4	7,882	98.0	86.4
1994	2.4	40.4	4.8	10.17	99.5	8,188	98.5	87.9
1995	2.4	41.0	4.8	10.33	99.5	8,895	98.6	89.4
1996	2.3	41.5	5.0	10.61	99.6	9,419	98.9	90.4
1997	2.3	41.8	5.2	10.43	99.6	9,905	99.3	91.0
1998	2.3	41.6	5.2	10.63	99.7	10,091	99.2	91.5
1999	2.2	41.5	5.4	10.83	99.7	9,885	99.2	92.1
2000	2.1	41.5	5.5	11.02	99.0	10,191	99.6	93.1
2001	2.0	41.8	5.7	11.09	99.7	10,415	99.7	93.6
2002	2.0	41.6	5.9	11.25	99.8	10,523	99.7	94.1
2003	1.9	40.9	6.2	11.38	99.7	10,820	99.8	94.7
2004	1.9	41.2	6.5	11.57	99.8	11,343	99.7	95.0
2005	1.9	43.5	7.5	11.70	99.8	11,863	99.8	94.9
2006	1.9	45.0	7.1	11.83	99.8	12,209	99.8	95.2
2007	1.8	44.9	7.2	12.09	99.8	13,556	99.9	95.2

† Average of schooling years for women in economically active age.

**Figure 4:**
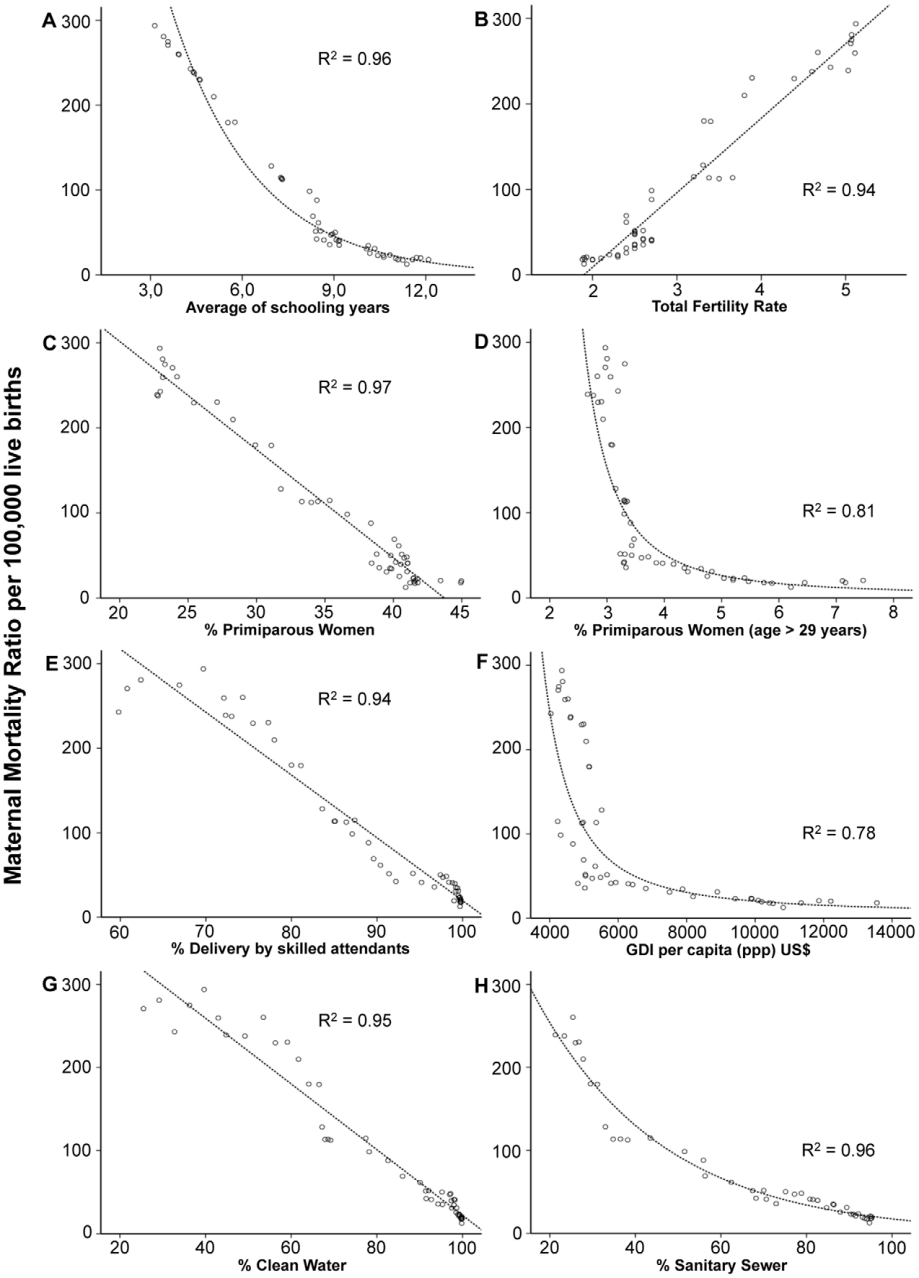
Correlations between parallel time series of maternal mortality ratio and different determinants, Chile 1957–2007. A strong (R^2^­>90) inverse correlation can be observed in charts A, C, E, G y H and a direct correlation can be observed in chart B (Total Fertility Rate). Correlations were slightly lower for charts D and F, both showing an inverse relationship.

In Figure 4, we explored the relationship between each independent variable and the MMR. Strong correlations were observed, and the goodness-of-fit of the curves with the maternal mortality trend were linear or exponential showing *R^2^* coefficient values between 0.78 (GDI per capita) and 0.97 (% of primiparous women giving birth) confirming that all these factors modified in parallel with the MMR.

### Segmented regression analysis

**Figure 5:**
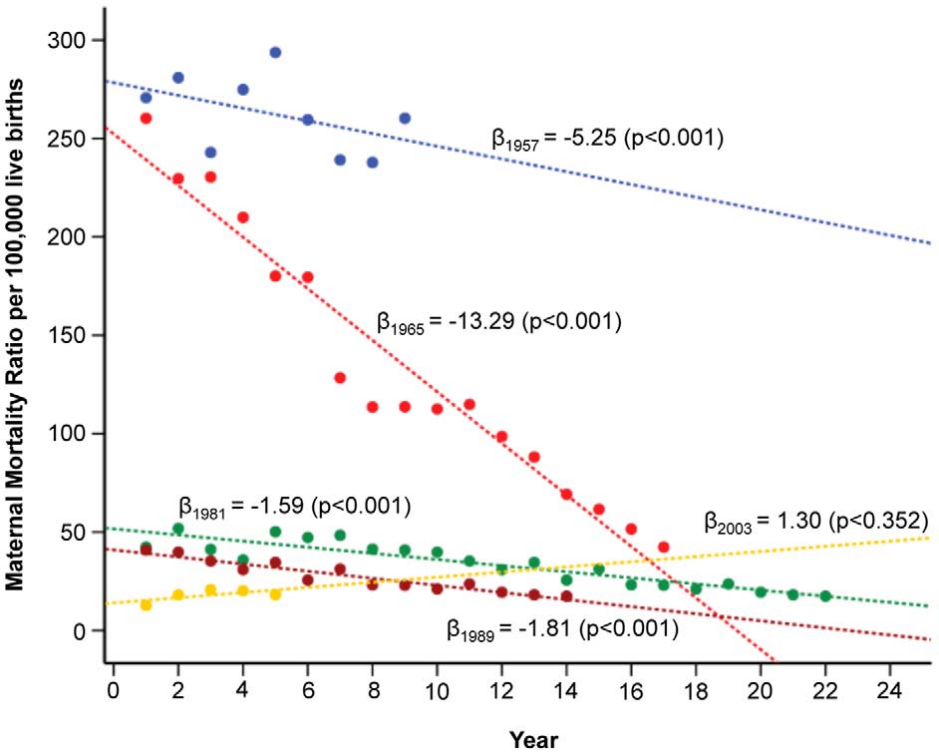
Slopes of different segments observed in the trend of the maternal mortality ratio between 1957 and 2007. The slopes for the periods 1981 to 2003 and 1989 to 2003 were parallel and no statistical difference was detected in β-coefficients.

In the segmented model, we considered the main join points representing historical changes (1965 and 1989) and other alternative segments. After 1965, we observed a continuous downward trend in the MMR until 1981, with a visual change in the slope from this point until 2003 in the scatterplot (Figure S3, Appendix S2). Thus, we sequentially incorporated these two segments in the initial model (Figure S4, Appendix S2). Figure 5 represents the slope decomposition for each segment, and its value (*β*-coefficient) matched on the same time scale in absolute years. After abortion became illegal in 1989, a decreasing trend in the MMR was observed, from 41.3 to 12.7 in 2003 (69.2% reduction). However, no evidence of a significant change in the slope from 1981 was observed. In fact, the slopes for the periods 1981 to 2003 and 1989 to 2003 were parallel and no statistical difference was detected in *β*-coefficients (Figure 5). Moreover, the AR component was 31% in the initial model and 33% in the final model excluding 1989 and 2003 (Table 2). Therefore, we used the initial slope from 1957 and the slopes related to the periods 1965 to 2007 and 1981 to 2007 to define the different time periods; the slope related to years 1989 to 2007, representing the period with abortion prohibition, did not influence the subsequent pathway regression modelling.

**Table 2:** Segmented regression models assessing different join points in the time series of the maternal mortality ratio from 1957 to 2007 in Chile.

Segments	Initial Model		Final Model	
	β	p-value	B	p-value
Initial slope from 1957	−5.23	0.028	−5.30	0.022
Join point 1965	14.93	0.218	15.33	0.195
Slope from 1965	−8.34	0.002	−8.21	0.002
Join point 1981	1.91	0.884	7.55	0.431
Slope from 1981	13.98	0.001	12.27	0.001
Join point 1989	−3.33	0.786	*ruled out*	*ruled out*
Slope from 1989	−2.24	0.394	*ruled out*	*ruled out*
Join point 2003	−3.05	0.843	*ruled out*	*ruled out*
Slope from 2003	3.32	0.469	*ruled out*	*ruled out*
*AR-component*	0.31	0.039	0.33	0.021
*Constant of the model*	283.73	0.001	283.84	0.001

### Pathway modelling

The pathway model utilized in this study incorporates a segmented regression model for adjusting for historical periods. The general specification of the model is as follows:
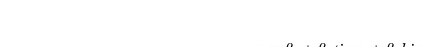



Every *β*-coefficient represents the corresponding slope for any variable in the model. *β_0_* is the constant or intercept of the model. *β_1_time_t_* estimates the average change in the outcome measures that occur each year during the study period since 1957. *β_2_historical.policy_t_* estimates the change in outcome measures immediately after an intervention or historical policy was implemented (*e.g*. educational policy and maternal health programs at year 1965), or after a join point was identified in the previous segmented regression model (*e.g.* join point of year 1981). *β_3_years.after.policy_t_* estimates the average change in outcome measures in the years after the historical policy intervention (*e.g.* 1965 or 1981). *β_4_education.years_t_* estimates the average change in outcome measures related to the women's education level. *Β_5_X_t_* represents *ceteris paribus* the average change in outcome due to an independent variable (*e.g.* percent of deliveries by skilled attendants, fertility rate, percent of sanitary sewer access, etc). Finally *e* ***_t_*** is a term for residual error and *y_t_* represents the outcome, in this time series study represented by the MMR (number of maternal deaths per 100,000 live births) at time *t*.

Results from the pathway modelling are presented in Table 3. The first model (model 1) is utilized to estimate the effect of every variable just controlling for the initial trend from 1957. In this case, we are not controlling by historical policies and education level.

**Table 3:** Pathway modelling using autoregressive integrated moving average (ARIMA) models for assessing the different predictors of the maternal mortality ratio in a time series from 1957 to 2007 in Chile.

Predictors	Model 1		Model 2		Model 3		Model 4	
	β	*p-value*	β	*p-value*	B	*p-value*	β	*p-value*
Average number of schooling years	−32.20	*0.001*	–	*–*	–	*–*	–	–
TFR	40.53	*0.001*	8.04	*0.328*	7.94	*0.344*	−0.33	*0.961*
Primiparous (%)	−10.01	*0.001*	−2.27	*0.139*	−3.32	*0.037*	0.52	*0.734*
Primiparous >29 years	8.09	*0.447*	28.29	*0.001*	30.09	*0.001*	4.30	*0.438*
Delivery by skilled attendants (%)	2.41	*0.135*	−2.41	*0.003*	−4.58	*0.001*	0.29	*0.758*
GDI per capita (PPP)	2.02	*0.729*	4.04	*0.237*	5.12	*0.145*	−1.03	*0.706*
Clean water (%)	−2.32	*0.002*	−1.98	*0.001*	−2.78	*0.001*	0.24	*0.765*
Sanitary sewer (%)	−1.57	*0.196*	−0.73	*0.319*	−2.16	*0.001*	0.17	*0.726*
Initial slope from 1957	−5.25	*0.001*	0.34	*0.724*	−1.76	*0.576*	−1.36	*0.348*
Join point 1965	25.79	*0.092*	4.15	*0.657*	–	*–*	5.28	*0.461*
Slope from 1965	0.87	*0.878*	2.22	*0.516*	–	*–*	−2.87	*0.086*
Join point 1981	−8.60	*0.475*	−22.11	*0.001*	−16.12	*0.014*	–	–
Slope from 1981	9.21	*0.001*	5.82	*0.001*	7.13	*0.001*	–	–

β is the regression coefficient or the estimate of the average change in the maternal mortality ratio per 100,000 live births per unit of change in the independent variable. GDI (Gross Domestic Income) refers to the Gross Domestic Product per 1,000 US dollars. The pathway modelling approach is as follows: Model 1 adjusted for the initial slope in 1957; Model 2 is Model 1 adjusted for the average number of schooling years; Model 3 is Model 2 additionally adjusted for the join point and the slope for the segment from 1965; and Model 4 is Model 3 adjusted for the join point and the slope for the segment from 1981.



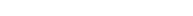



The second model (model 2), adding the antecedent variable (women's education level) is:
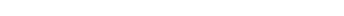



The third model (model 3), adding the historical period 1965 the years after it is:
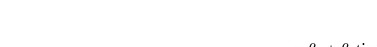



The final model (model 4), adding the joint point of 1981 and the years after it is:
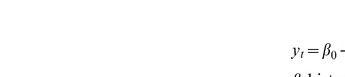



In Table 3, the change in *β*-coefficient represents the increasing or decreasing effect on the MMR for each unit of increment in the independent variable. The initial effects of the TFR and the percent of primiparous women on the MMR were attenuated after adjusting for education (model 2) and were not substantially changed in the following models. The null effect of the percent of primiparous women >29 years showed a positive increase in models 2 and 3 but was substantially attenuated after controlling for the segment trend from 1981 in model 4. After 1965, for every 1% increment in the percent of women giving birth who were primiparous and greater than 29 years of age, there was an increase of 30 maternal deaths per 100,000 live births. An inverse relationship between the MMR and the percent of deliveries by skilled birth attendants was observed in model 2, which increased after 1965 (model 3). We estimated that there was a decrease of −4.58 maternal deaths per 100,000 live births for each 1% increment in the number of deliveries performed by skilled attendants. This effect disappeared after 1981. No effect was observed for GDI per capita. The percent of women with access to clean water was inversely related to the MMR, increasing from −1.98 (model 2) to −2.78 per 100,000 live births after controlling for the trend from 1965 (model 3). This relationship disappeared after 1981 (model 4). The percent of women with sanitary sewer access was also inversely related to the MMR, but only between 1965 and 1981, with a decrease of −2.16 maternal deaths per 100,000 live births for each 1% increment. Finally, controlling simultaneously for all predictors, the average number of years of female education remained a significant predictor throughout the entire MMR trend. For each additional year of education for women of reproductive age, the reduction in the MMR was estimated to be −29.31 per 100,000 live births.

## Discussion

**Figure 6:**
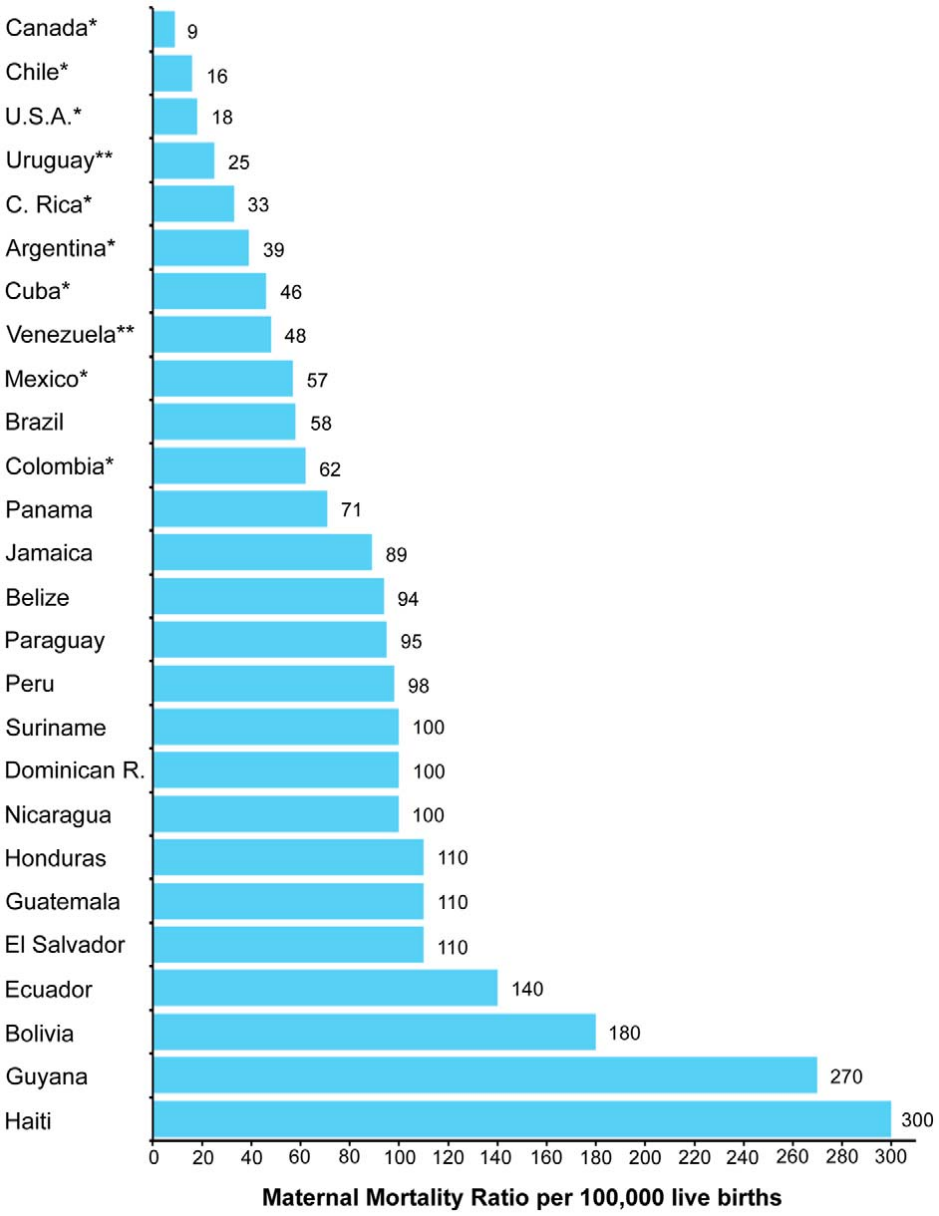
Ranking of maternal mortality ratios (MMR) in the American continent for 2008. Official MMR for Chile [45] is compared to official World Health Organization (WHO) estimates [35] on other American countries, except for those with asterisks. *Official domestic data for Canada [43], [44], Chile [45], United States [46], [47], Costa Rica [48], Cuba [49], Argentina [50], Mexico [51], [52] and Colombia [53], [54]. An important overestimation for these countries is observed when comparing WHO estimates to official domestic data. For instance, according to official data available for the U.S., 795 maternal deaths [47] and 4,247,694 live births [46] occurred in 2008. In consequence, the MMR for the U.S. that year was 18.7 per 100,000 live births. For Chile, the MMR was 16.5 per 100,000 live births (41 maternal deaths and 248,366 live births [45]). The same figure, but using indirect estimates for MMR reported by WHO [35] was 24 per 100,000 live births for the U.S. and 26 per 100,000 live births for Chile. In consequence, there is an overestimation of 28.3% for the U.S. and 57.6% for Chile in the WHO report. ** Data extracted from the study by Hogan *et* *al.* [3]

The findings of this study confirm that the MMR in Chile has steadily and consistently decreased, reaching the lowest rate in Latin America and the second lowest rate in the American continent when the indirect estimates from the World Health Organization [35] are replaced by the official figures observed for Canada [43], [44], Chile [45], United States [46], [47], Costa Rica [48], Cuba [49], Argentina [50], Mexico [51], [52] and Colombia [53], [54] in 2008 (Figure 6; data calculation is presented in Table S7, Appendix S2). Although the descending trend was continuous until 2003, the year 1965 representing the implementation of educational and maternal health policies had a significant impact on the mortality trend. The segmented regression identified two clear changes in the slope of the decline from 1957 to 2007. Between 1965 and 1981, there was an accelerated reduction of 84% in the MMR of approximately −13.29 per 100,000 live births each year (*rapid phase*). Between 1981 and 2003, the slope became less pronounced, at −1.59 per 100,000 live births each year (*slow phase*). All mortality causes declined in parallel, but the proportion of maternal deaths due to indirect causes (Figure S1) increased during the slower phase. Abortion was the leading cause of maternal death between 1957 and 1989, decreasing progressively between 1981 and 2007 (Figure S2). The reduction in the MMR was not related to the legal status of abortion –*i.e.* the prohibition of therapeutic abortion. Our analyses suggest that women's education level and several other factors worked synergistically to explain the decrease in the MMR observed in Chile, particularly during the rapid phase.

### Abortion legislation

Abortion is a very complex issue, which appears confounded by many factors; however, the actual effects of legal restrictions on maternal health have virtually remained unexplored. At the present time, some researchers and policymakers postulate that legal restriction of abortion is one of the main determinants of maternal mortality in developing countries, mainly based on indirect evidence related to purportedly high proportion of all maternal deaths, or due to indirect estimates of the number of maternal deaths and clandestine abortions [22], [24]–[26], [55]–[59]. It is generally thought that laws strongly restricting abortion should result in increasing maternal mortality and morbidity because of an increase in the number of unsafe abortions. This rationale states that legalisation or decriminalisation of abortion is an appropriate strategy to decrease maternal mortality, allowing access to safe, legal abortions [22], [23], [25], [26], [55], [57]–[59]. The validity of this assumption depends on whether the legal status of abortion is causally associated with the prevalence of illegal abortion, the safety of the abortive procedure, and maternal morbidity and mortality exhibited in general. Nevertheless, no direct evidence testing this causal assumption in developing countries currently exists. Furthermore, the lowest MMRs observed in European countries such as Ireland, Malta and Poland [3], [35], where abortion is severely restricted by law, suggest that this assumption may be untrue.

After 1989, Chile is recognised as one of the countries with the most restrictive abortion laws in the world and has been criticised because of the purported possible deleterious consequences on maternal health [60], [61]. Nevertheless, the present study provides counterintuitive evidence showing that making abortion illegal is not necessarily equivalent to promoting unsafe abortion, especially in terms of maternal morbidity and mortality [34]. Chile's abortion prohibition in 1989 did not cause an increase in the MMR in this country. On the contrary, after abortion prohibition, the MMR decreased from 41.3 to 12.7 per 100,000 live births –a decrease of 69.2% in fourteen years. Excluding ectopic pregnancy, the absolute risk of death due to unspecified abortion is one in two million women at fertile age. Our study indicates that improvements in maternal health and a dramatic decrease in the MMR occurred without legalization of abortion. This does not imply that there are no illegal or clandestine abortions in Chile. Rather, current abortion mortality ratio and recent epidemiologic studies of abortion rates in this country [62]–[64] suggest that clandestine abortion may have been reduced in parallel with maternal mortality and may have currently reached a steady state based on stable ratios between live births and hospitalizations by abortion. It is expected that any major increase in the magnitude of clandestine abortions should be necessarily followed by an increase in abortion hospitalizations [34], [64]. For example, in 1960, when the leading cause of mortality was abortion, there were 287,063 live births and 57,368 hospitalizations from abortion (whether spontaneous or induced), representing a 5∶1 ratio [65]. In the last decade, the ratio between live births and hospitalizations from abortion has remained relatively stable at approximately 7∶1 (Table S8, Appendix S2). Consequently, it can be suggested that the total number of abortions (whether spontaneous or induced) have not substantially increased [62]–[64], [66].

Although Shepard and Casas Becerra [61] state that “[m]ore than 99% of induced abortions are not reported at all, disguised as a different procedure or reported as spontaneous abortion in public hospitals” this speculative assumption is, however, unsupported by current epidemiological studies on abortion in Chilean hospitals [34], [62]. In fact, a recent epidemiological study [64] based on the Chilean official hospitalization registry and the biological probabilities of viable conception [67] and clinical spontaneus miscarriage [68], [69], estimated that 12% to 19% out of all abortions registered in hospitals may be related to induced abortions from 2001 to 2008 (Table S8, Appendix S2). Furthermore, nowadays there is no reason to support that abortion diagnosis using ICD-10 (*e.g.* using the code O06 “unspecified abortion”) is misreported or intentionally misclassified by medical doctors in Chilean hospitals; professional secret and patient confidentiality rights currently protect them. On the contrary, they are exposed to legal sanctions if intentionally lie or falsify any diagnosis as cause of death.

It is well documented that the Chilean program providing contraceptive methods after clandestine abortion was effective in decreasing abortion rates [34], [41], [63], [70]. In addition, the methods used to conduct clandestine abortions at present may have lower rates of severe complications than the methods used in the 1960s, mainly based on highly invasive self-conducted procedures [34], [41], [55], [62], [63], [65]. Therefore, the practically null abortion mortality observed in Chile nowadays can be explained by both a reduced number of clandestine abortions and a lower rate of severe abortion-related complications [64]. This phenomenon also seems to be related to joint-effects between increasing educational level and changes on the reproductive behaviour of Chilean women, an observation that requires further research.

### Fertility and reproductive behaviour

It has been proposed that fertility is an important determinant of reduced maternal mortality [3], [5]–[9], [71]. This has been explained by a theoretically lower obstetric risk associated with fewer pregnancies during a woman's lifetime. For instance, in a recent study it was estimated that about 39 percent of the decline in the MMR in Bangladesh was attributable to fertility decline between 1990 and 2008, compared with 32 percent in India and 22 percent in Pakistan [7]. Nevertheless, that study did not control for major third variables such as the change in women's educational level. In Chile, we observed a strong correlation between the MMR, TFR, the total percent of primiparous women and the percent of primiparous women >29 years giving birth (Figure 4, B, C and D). After adjusting for educational level, however, TFR did not have an important impact on the decreasing maternal mortality trend. In contrast, the increase in the number of first pregnancies at advanced ages was directly associated with the MMR, during the rapid phase of the maternal mortality reduction. For every 1% increment in primiparous women giving birth older than 29 years of age, an increase of 30 maternal deaths per 100,000 live births was estimated (Table 3). This finding indicates the presence of a ‘fertility paradox’: when TFR decreases and produces a delayed motherhood it can also provoke a deleterious effect on maternal health via an increase of the obstetric risk associated with childbearing at advanced ages. This remains consistent with the results of recent studies in developed nations [72]–[75].

In Chile, TFR decreased from 5.0 to 1.88 from 1957 to 2007 in the female population, the percent of primiparous women giving birth increased from 23.9% to 44.9%, and the percent of primiparous women >29 years of age increased from 3.0% to 7.2% respectively (Table 1). Altogether, these factors indicate an important shift in the reproductive behaviour of the population, providing evidence that Chilean women are enabled to control their own fertility, delaying motherhood and decreasing the size of the family without greater adherence to artificial contraceptives than developed nations. Although the primary care system currently provides universal access to a variety of contraceptive methods, actual use rate of hormonal contraceptives and intrauterine devices in Chile reaches approximately 36% of women at reproductive age [76]. Therefore, as in developed nations [77], other factors not limited to the use of artificial contraceptives seem to be contributing to the reduction in TFR in Chile. One such factor could be women's increasing level of education.

Interestingly, when the number of years of education of the female population is included in the explanatory model, the strong correlation between TFR and maternal mortality reduction is substantially attenuated, suggesting that women's education level may simultaneously influence both the MMR and TFR. Without increasing women's education level, the simple availability of maternal health facilities, medicines and skilled personnel may be insufficient to improve maternal health [12], [13]. In this sense, increasing education levels would be related to a higher knowledge favouring the utilization of available maternal health facilities. In addition, education promotes higher autonomy to women, allowing them to take the control of their own fertility using the method for fertility regulation of her preference.

### Maternal health facilities

There is wide scientific consensus that delivery by skilled birth attendants is a key factor in reducing maternal mortality and constitutes one of the main strategies to reach MDG-5 [17]–[21], [27], [78], [79]. The findings of this Chilean natural experiment confirm this consensus. The impact of delivery by skilled attendants was stronger during the rapid phase of maternal mortality reduction, and this effect was increased by women's educational level. The percent of deliveries attended by a skilled birth attendant increased from 60.8% in 1957 to more than 90% by 1980 (Table 1), reflecting the impact of the primary care maternal health programs on early prenatal diagnosis and opportune derivation for delivery in the secondary system. Currently, over 99% of all deliveries occur in hospitals or maternities and this figure was reached in 1999.

In 1964, the main objective of the maternal health program was to provide universal access to maternal healthcare services, including early pregnancy diagnosis (before 12 weeks), thus increasing the number of women enrolled into prenatal care early in pregnancy. This initiative was the latest development of the maternal-child health programs that began in 1937 with the promulgation of the ‘Mother-Child law’ [80]. Complementary nutrition programs for pregnant women and their children, initiated immediately with the promulgation of this law, were reinforced mainly by distributing fortified milk at primary care health centres. The distribution of milk at primary care clinics brought mothers into the primary care clinics, creating new opportunities for prenatal, perinatal and postnatal care for both mother and child [81], [82]. Simultaneously, this strategy practically eradicated malnutrition, increased birth weight and contributed to the noteworthy reduction in infant mortality observed in Chile –in which stands today at 3.1 per 1,000 live births for infants aged 28 days to 1 year [83].

Nevertheless, no additional impact on the MMR trend by the percent of deliveries by skilled birth attendants was seen after 1981, during the slow phase of maternal mortality reduction. Other factors, such as access to emergency obstetrical units and the development of specialised diagnostic centres for high-risk pregnancies, appear to have been associated with reductions in maternal mortality as well [19], [20]. Although this study did not directly control for those variables, previous research has shown that Chile experienced a significant expansion in access to emergency and specialised obstetric care since 1990, a fact that could explain the subsequent decrease in maternal mortality [63], [78].

It is of note that the reduction in the absolute number of maternal deaths and the general MMR was continuous until 2003. Nevertheless, a change in the types of maternal deaths during the slow phase was observed, during which the proportion of deaths due to hypertension, eclampsia and toxaemias increased (Figure 2, B and C). Furthermore, since 1990 a substantial change occurred during which the proportion of indirect causes increased from 8.6% to 33.2% while abortion decreased from 29.2% to 6.2% (Figure 2, C and D). This change suggests the apparition of a more complex residual pattern of maternal morbidity which required specialized, immediate medical services for decreasing the MMR trend. According to the most recent report published by INE [84], the MMR for 2009 was 16.9 per 100,000 live births (43 deaths) and the figures of indirect causes (codes 099, 098), gestational hypertension and eclampsia (codes 014, 015), abortion (code 006), and other direct obstetric causes were 18 (41.9%), 11 (25.6%), 1 (2.3%) and 13 (30.2%) respectively.

A plausible explanation for this phenomenon is provided by our time series on reproductive variables (Table 1). It can be observed that an important change occurred in the reproductive pattern characterized by a rapid reduction of TFR from 5.0 to 2.5 between 1957 and 1985, representing 80% out of the total observed reduction. Similarly, 79% out of total increase for primiparous women giving birth was produced during this period. In direct contrast, just 9.5% out of the entire increase from primiparous women >29 years (a direct indicator of pregnancy at an advanced reproductive age) occurred between 1957 and 1985. In other words, 90.5% of this change concentrated between 1985 and 2007 indicating an accelerated transition to a delayed motherhood. This reproductive pattern is known to be consistently associated to increased obstetric mortality and morbidity by pre-existing chronic conditions, obesity, gestational hypertension, diabetes, eclampsia, third trimester haemorrhage, caesarean section among others [72], [85]–[88]. Epidemiological studies conducted during the last decade in Chilean pregnant women [89]–[91] have established that the first cause of maternal morbidity and death is related to non-obstetric pre-existing chronic conditions –*i.e.* indirect causes, code O99– occurring preferentially in pregnant women of an advanced reproductive age. In contrast this population exhibits null mortality by abortion [91]. Childbearing at advanced ages emerged progressively in Chile from 1985 and it continues to rapidly increase, probably hindering further reductions in maternal mortality trends.

Finally, the two phases observed in the slope of the decrease in maternal mortality suggest the presence of a threshold in the transition toward this pattern, at which access to primary care facilities and delivery by skilled attendants no longer suffices to reduce maternal mortality and an expansion and opportune referral to specialized medical services is required. In the case of Chile, this threshold in the MMR appears to have been situated between 40 and 50 deaths per 100,000 live births along with a TFR of 2.5 –*i.e.* the figures observed between 1981 and 1985 in this country. Whether the presence of this transitional threshold is a reproducible observation in other developing countries requires further research.

### Women's education level

Our findings are consistent with recent research showing that the education level of the female population is strongly correlated with the MMR [11], [14], [15], [30], [71]. Moreover, the pathway regression modelling utilised in this study provides evidence that the observed decrease in maternal mortality has been partially a function of the education level of women, which simultaneously *modulates* the effects of other variables including reproductive behaviour and access to maternal health facilities. For example, besides the ‘fertility paradox’ discussed above, the decreasing effect of the percent of delivery by skilled attendants over the MMR increased by 90% (*i.e.* from −2.41 to −4.58 per 100,000 live births of reduction for each 1% increment in the number of deliveries performed by skilled attendants) after adjusting for women's education level in the rapid phase of mortality reduction (Table 3).

In 1965, a law regarding free and mandatory primary education up to a minimum of 8 years was implemented in Chile, which for all practical purposes resulted in a sustained and rapid increase in public school enrolment. The average number of years of female education increased rapidly from 3.1 to 12 years between 1957 and 2007. Increasing the number of schools facilitated access to free preventive health services for children and their families in the growing public health primary care network (urban primary care centres and rural units). Health care services were provided by physicians, nurses, professional midwifes, and trained paramedical personnel. This phenomenon resulted on an important social shift in Chile, which contributed to the development of social support networks between women and families who now shared a novel opportunity to access education for their children. The complementary school nutrition program initiated in the 1950s increased its coverage after 1965, providing breakfast and lunch to all newly eligible school children [40]. This change allowed more work opportunities because Chilean women had progressively more available time not devoted to taking care of children, thus increasing family income and education levels.

Our findings can be interpreted in several ways. Education may represent acquired knowledge and skills which enable an individual to manage the social system to meet desired ends [92]. In life course epidemiology, education is considered to be a measure of both early life circumstances (as the opportunities available to an individual are likely to be patterned by parental social class), and future socioeconomic trajectory [93]. Therefore, education may have an ‘antecedent’ role to economic development indicators, such as income, clean water supply, sanitary sewer access and other environmental variables [31]. Furthermore, education promotes increasing autonomy, awareness, responsibility and knowledge for self-care, healthy lifestyles and behaviours [13], [71], [92]. This ‘education hypothesis’ explains the direct and indirect effects on maternal mortality reduction, as education likely promotes the efficient utilization of maternal and reproductive health facilities [12]–[15], [30], [71], including access to early antenatal care, opportune perinatal and postnatal care, artificial contraception, natural family planning methods or postponing marriage. However, a longer duration of female education may enhance participation in the workforce and encourage women to control their own fertility and postpone excessively motherhood which may paradoxically increase complications and maternal deaths for pre-existing chronic conditions or problems such as gestational diabetes or hypertension.

### Limitations

To monitor the progress in decreasing maternal mortality and to guide the decision-making process, it is fundamental for countries to have data that enables them to estimate the MMR in their population, evaluate the MMR time trend, estimate the main causes of maternal death; and, where possible, evaluate the impact of different determinants which could positively or negatively influence maternal mortality [2]. Nevertheless, the paucity of reliable, large, continuous and parallel time series data in developing countries makes this task difficult [32], [33], [94]. This problem has been satisfactorily addressed in this study because of the reliability and extensive availability of parallel time series of official data over the past fifty years in Chile. Considering the strict protocol for active epidemiological surveillance on maternal and infant mortality registry implemented in the early 1980s, it is unlikely that the observed reduction could be explained by unobserved illegal abortion deaths or misclassification for other causes. Currently, any maternal death occurring in Chile is audited by the sanitary authority revising the clinical registries, interviewing the relatives, and the medical personnel under strict confidentiality rules for determining the primary cause of death. Nevertheless, we cannot conduct an exact homologation of the four ICD versions used in Chile over the last fifty years (Appendix S1); a study of specific causes is for this reason limited. For instance, it was not possible to accurately separate spontaneous and induced abortion deaths for all the periods and therefore these statistics were combined. In addition, we can not to accurately identify indirect causes using the ICD-7 and ICD-8 in the Chilean registry and therefore, we just present data for indirect causes from 1980.

Although this study is a natural experiment based on time series, and therefore does not allow us to establish causal relationships, we incorporated robust segmented regression techniques to test our hypotheses. The use of large time series can be an excellent method for conducting naturalistic studies of the effects of changes at a systems level, which capitalises on existing data, allows intuitive graphical representation, estimates trends before and after an intervention, and takes into account programmatic and legal variables, such as obligatory schooling, maternal health programs, family planning, and abortion legislation [42], [95], [96]. In addition, effect sizes can be estimated at different times after the interventions, simultaneously controlling for other variables using parallel time series to take advantage of multiple causal pathway modelling. If a specific event or moment in time is a good proxy for method by which intervention and control groups were assigned (*i.e.*, trends before and after interventions), natural experiments based on interrupted time series can be robust alternatives to classical randomised experiments insofar as external validity is concerned, especially if the natural experiment is a general population-based study minimising both individual and ecological fallacies [32], [97], [98].

Collinearity is another potential problem when multiple parallel time series are used to estimate unbiased effect sizes in multiple regression analyses. The variables considered in this study all followed parallel decreasing or increasing trajectories with respect to the maternal mortality trend. In fact, we observed high correlations between every predictor and MMR and high correlations between each predictor and time (Figure 4). The ordinary least squares method therefore would not have been an appropriate technique for our large parallel time series. However, this problem is controlled for by default, in the autoregressive component of the ARIMA models, and it can be additionally addressed using segmented regression. For example, it was clear using this robust analytical approach that the impact of several parallel predictors on the MMR varied over time, and this result was confirmed in sequential models incorporating the join points of 1965 and 1981. By using segmented regression we demonstrated that there were in fact two phases in the downward mortality trend, ruling out other possible causes for changes in the trend due to intuitive observation or objective historical antecedents such as the change in abortion legislation. In addition, pathway modelling allowed us to assess specifically how changes in women's educational level modulated the effect of other important factors, suggesting the presence of synergistic effects.

### Conclusions

Taken together, the Chilean natural experiment over the last fifty years suggests that the progress on maternal health in developing countries is a function of the following factors: an increase in the educational level of women, complementary nutrition for pregnant women and their children in the primary care network and schools, universal access to improved maternal health facilities (early prenatal care, delivery by skilled birth attendants, postnatal care, availability of emergency obstetric units and specialized obstetric care); changes in women's reproductive behaviour enabling them to control their own fertility; and improvements in the sanitary system –*i.e.* clean water supply and sanitary sewer access. Furthermore, it is confirmed that women's educational level appears to have an important modulating effect on other variables, especially promoting the utilization of maternal health facilities and modifying the reproductive behaviour. Consequently, we propose that these strategies outlined in different MDGs and implemented in different countries may act synergistically and rapidly to decrease maternal deaths in the developing world.

On the other hand, a change in the types of maternal deaths appeared progressively in Chile between 1985 and 2007 increasing the proportion of deaths due to hypertension, eclampsia, and toxaemias and especially related to pre-existing chronic conditions over the last decade –*i.e.* indirect causes of maternal death. The residual pattern of maternal mortality in Chile has been very difficult to address, requiring an important expansion of emergency units and specialized obstetric services. This phenomenon appears to be explained by an accelerated change in the reproductive pattern characterized by low fertility rate, delayed motherhood and an increased proportion of pregnancies occurring at an advanced reproductive age. Finally, prohibition of abortion in Chile did not influence the downward trend in the maternal mortality ratio. Thus, the legal status of abortion does not appear to be related to overall rates of maternal mortality.

## Supporting Information

Appendix S1 Methodological complements.
(PDF)

Appendix S2 Complementary results.
(PDF)

Figure S1 Relative importance of maternal deaths by indirect causes (percent from all maternal deaths including abortion) based on 24 continuous five-year intervals between 1980 and 2007 observed in Chile. The intersection (white circle) of the broken-lines identifies the interval 1991–1995 as the point in which the percent of indirect causes of maternal death began to progressively increase beyond the limit of 10%. During the period of analysis the International Classification of Diseases codes version 9th (1980 to 1996) and 10th were used (1997 to present).
(TIF)

Figure S2 Relative importance of death by abortion (percent from all direct and indirect maternal causes of death) based on 47 continuous five-year intervals between 1957 and 2007 observed in Chile. The intersection (white circle) of the broken-lines identifies the interval 1981–1985 as the point in which the percent of abortion deaths began to progressively decrease under the limit of 35%. During the period of analysis the International Classification of Diseases codes version 7th (1957 to 1967), 8th (1968 to 1979), 9th (1980 to 1996) and 10th were used (1997 to present). The percents were calculated considering all deaths by spontaneous and induced abortions combined but excluding ectopic pregnancy, hydatidiform mole and other abnormal products of conception.
(TIF)

Figure S3 Scatterplot identifying possible join points on maternal mortality trend in Chile for segmented regression analyses.
(TIF)

Figure S4 Proposed segmented regression model applied to the Chilean time series from 1957 to 2007.
(TIF)

Table S1 International Classification of Diseases (ICD) 7th–10th versions for classifying maternal death causes in Chile. The groups for homologation were selected from ICD 7th version, list A.
(PDF)

Table S2 International Classification of Diseases (ICD) 7th version for classifying maternal death causes in Chile.
(PDF)

Table S3 International Classification of Diseases (ICD) 8th version for classifying maternal death causes in Chile. Homologation with five groups from ICD 7th version, list A.
(PDF)

Table S4 International Classification of Diseases (ICD) 9th version for classifying maternal death causes in Chile. Homologation with five groups from ICD 7th version, list A.
(PDF)

Table S5 International Classification of Diseases (ICD) 10th version for classifying maternal death causes in Chile. Homologation with five groups from ICD 7th version, list A.
(PDF)

Table S6 Time series of maternal deaths and selected indicators, Chile 1957–2007.
(PDF)

Table S7 Direct comparison of the maternal mortality ratio (MMR) estimates by the World Health Organization (WHO) report with available official domestic data in eight countries of the American continent in 2008. These countries are classified in list A according to the completeness of their vital statistics by United Nations (*i.e.* civil registration of deaths is virtually complete). An important overestimation (*e.g.* 35.4% for Colombia, 48.6% for Mexico, 57.6% for Chile and 76.3% for Argentina) can be observed in WHO maternal mortality estimates for these countries with full official records of maternal deaths. In consequence, progress in maternal health seems to be underestimated in several Latin American countries by WHO technical reports.
(PDF)

Table S8 Abortion hospitalizations in Chile based on official data and estimated proportions for clinical spontaneous abortions and clandestine induced abortions for the period 2001–2008. During this period, hospitalizations from clandestine abortion complications are estimated between 12% and 19% out of total hospitalizations by abortion (calculated as the difference between expected hospitalizations by clinical spontaneous abortion and observed hospitalizations by abortion; for additional information, see Appendix S2).
(PDF)
