# Supplementary material for: Women's Education Level, Maternal Health Facilities, Abortion Legislation and Maternal Deaths: A Natural Experiment in Chile from 1957 to 2007
Source: PLoS One. 2012 May 4;7(5):e36613. doi: 10.1371/journal.pone.0036613 (PMC3344918; doi:10.1371/journal.pone.0036613)
Supplement: Appendix S1 — Methodological complements. (PDF) [file pone.0036613.s001.pdf]

APPENDIX S1
METHODOLOGICAL COMPLEMENTS

Maternal death registry

According to an analysis of official vital statistic yearbooks that have been published continuously since 1957 by the Chilean National Institutes of Statistics (INE), we identified four well-defined periods of registry based on international codes of diseases (ICD) used to classify the causes of death in the country. During the first period, from 1958 to 1967, the causes of maternal mortality were classified according the ICD-7 (7th version)[1], including codes A115, A116, A117, A118, A119 and A120. Codes A118 and A119 corresponded to abortion deaths (abortion without and with sepsis, respectively). In 1957 the ICD-6 (6th version) was utilized but the maternal deaths were directly homologated with ICD-7. During the second period, from 1968 to 1979, maternal deaths were classified using the ICD-8 (8th version)[2], specifically codes A112, A113, A114, A115, A116, and A117, using codes A114 (abortion induced by legal indications) and A115 (other and unspecified abortion) to characterize the total number of abortion deaths. From 1980 to 1996, the ICD-9 (9th version)[3] was used, and maternal deaths were classified using codes 630 to 676. Abortion deaths were represented by codes 632 (missed abortion), 634 (spontaneous abortion), 635 (legal induced abortion), 636 (illegal induced abortion), 637 (unspecified abortion), 638 (attempted but failed abortion), and 639 (complications following abortion and ectopic and molar pregnancies). The study did not consider codes 630 (hydatidiform mole), 631 (Other abnormal products of conception) and 633 (ectopic pregnancy), present in the ICD-9, as death caused by abortion, mainly because abortion is not the primary cause of death. Thus, these causes of death were individually considered. Finally, the ICD-10 (10th version)[4] has been used since 1997, including codes O00 to O99 to classify maternal deaths. During this period, the number of abortion deaths was computed according to codes O03 (spontaneous abortion), O04 (medical abortion), O05 (other abortion), O06 (non-specified abortion), O07 (failed abortion), and O08 (complications secondary to abortion). Similar to ICD-9, codes O00 (ectopic pregnancy), O01 (hydatidiform mole) and O02 (Other abnormal products of conception) were considered individually. No missing data were present in this time series.

Live births

To compute the maternal mortality ratio (MMR) in the statistical analyses – the number of women dying per 100,000 live births – we used the official time series of live births published by INE. For the annual registry of vital statistics from 1957 to 1967, the number of live births was directly corrected based on 95% registry integrity [5]. From 1968 to 1974, the data were once more directly corrected using a new study reporting 91.1% registry integrity [6]. However, according to a more recent study conducted in Chile [7], there was an increase in the delayed inscription of births with oscillations from 8% to 17% between 1957 and 1979. From 1980, the delayed inscription fell continuously from 12% to the present value of less than 1%. Thus, to serve the purposes of this study, the number of live births for every year was corrected using the delayed registration method, which allows a more precise and robust correction because it is self-generated and works using the number of delivered births during a certain year enrolled up to seven years later. No missing data were present in this time series.

Parallel time series

Women's education level was assessed using the construction of a parallel time series assessing the average years of schooling. In the absence of a single measure during the last fifty years, data provided by three official sources were used to obtain a single and complete time series. First, for the period from 1957 to 1999, we used the series published by the Central Bank of Chile (Banco Central de Chile) [8] and by the Economic History and Cliometrics Laboratory [9]. Additionally, we considered

data of general population surveys provided by the Central Bank regarding the average years of schooling from 1960 to 1999. These data are based on consecutive cross-sectional National Surveys of Employment over the economically active population conducted from October to December of each year. Second, for the period from 1980 to 2007, we also considered data estimated using the Social Characterization Survey (CASEN) conducted by the Chilean Ministry of Planning regarding the average years of schooling for economically active women. Finally, we considered the percentage of women with 9 or more schooling years at the moment of delivery published by INE in the annual registry of live births from 1957 to 2007. These variables were highly correlated and collinear (regression coefficient 0.97, $p<0.001$), validating the suitability of the estimation by a single time series of education level of the female population of fertile age. To obtain a set of 51 points representing the average number of schooling years from 1957 to 2007, we used a single imputation method based on multiple regressions, including all of the variables mentioned above. Multiple imputations were unnecessary [10] because the combined time series did not have missing values. In fact, in an exploratory analysis, the convergence was complete, and differences in the three generated multiple datasets were negligible. Therefore, a single imputation was appropriate to obtain the complete time series for the average of female schooling years. The imputation procedure has been described elsewhere [11], and it was conducted using the software Amelia II version 1.2 using the R statistical program.

Other independent predictors analyzed in continuous and parallel times series were income per capita, clean water supply, sanitary sewer coverage, total fertility rate (TFR), birth order (primiparous), and delivery by skilled attendants. For income, we used a complete time series of the Gross Domestic Income (GDI) at purchasing power parity (PPP) per capita in US dollars [12]. This measure represents the value of all final goods and services produced within a nation in a given year divided by the average population for the same year. The use of a PPP basis instead of a nominal basis approach is arguably more useful when comparing differences in living standards among nations because PPP takes into account the relative cost of living and the inflation rates of each country rather than using just the exchange rates, which may distort income real differences.

Additionally, we used continuous time series to analyze the clean water supply and sanitary sewer coverage for the entire country, expressed as percentages. These time series data were published by the Central Bank of Chile for the period between 1963 and 1999 [8], by the Chilean Superintendence of Sanitary Services (SISS) for the period between 1965 and 2009 and by Valenzuela and Jouravlev from 1950 [13,14].

Finally, complete time series for TFR (the average number of children that would have been born to a woman over her lifetime if she had experienced the exact age-specific fertility rates through her lifetime), the order of birth and the percentage of deliveries performed by skilled attendants (calculated in Chile as live births delivered by medical professionals in hospitals or maternities over total live births registered each year) were extracted from vital statistics yearbooks that have been published continuously since 1957 by INE. In the present study, TFR is considered to be a result of the overall factors influencing the reproductive behavior of the entire female population over time. On the other hand, the percentage of deliveries performed by skilled attendants is considered to include giving necessary supervision, care, and advice to women during pregnancy, labor, and the postpartum period; to conduct deliveries on their own; and to care for newborns.

Historical background

In this natural experiment, we additionally considered three historical interventions to influence women's health programs and, consequently, the MMR trend. The first of

these is the promotion and implementation of a law regarding free and mandatory education of a minimum of 8 years in 1965, which for all practical purposes resulted in a sustained and rapid increase in public school enrolment. The increasing number of schools facilitated access to free preventive health services for children and their families in the growing public health primary care network (urban primary care centers and rural units named "postas rurales", including health care provided by physicians, nurses, professional midwifes, and trained paramedical personnel). This phenomenon resulted on an important social shift in Chile, which contributed to the development of social support networks between women and families who shared a novel access opportunity to education for their children. Moreover, this change allowed more work opportunities because women had more available time not devoted to taking care of children, thus increasing family income and improving low education levels. The complementary school nutrition program initiated in the 1950s increased its coverage after 1965, providing breakfast and lunch to all newly eligible school children [15].

The second important phenomenon observed was a widespread maternal public health program along with a family planning program, both implemented between 1964 and 1967. The main objective of the maternal health program was to provide universal access to maternal healthcare services, including detection of early pregnancy (before 12 weeks), thus increasing access to prenatal control, which had been developing since 1937 with the promulgation of the "Mother-Child law" by the Minister of Health Dr. Eduardo Cruz-Coke [16]. Complementary nutrition programs for pregnant women and their children, initiated in 1937, were reinforced mainly by distributing fortified milk at primary care health centers. As Monckeberg stated, milk distribution at primary care clinics brought them closer to the mother's routine, creating a new instance for control and preservation of prenatal and postnatal health in both the mother and her children [17,18].

In the 1960s, hospitalizations for induced abortion complications were high in Chile [19-21]. For example, in 1960, the number of live births was 287,063, and the number of hospitalizations from abortion (whether spontaneous or induced) was 57,368 [19]. In 1995, these same figures were 275,760 and 28,981, respectively. In the last decade, the ratio between live births and abortions has remained relatively stable, and hospital discharges after abortions have decreased [22]. In contrast, epidemiological studies in 1960s suggested that a history of induced abortion was present in 22% of Chilean women between 20 and 44 years old. A history of induced abortion was concentrated in a group of women who tended to repeat the procedure three or more times in subsequent pregnancies, mainly married poor women of working class with three or more children and minimal or no education [19,21]. Only one-third of induced abortions were performed by medical personnel, and most of the complications were the result of self-induced abortions or clandestine abortions performed by unqualified individuals [19]. Between 1964 and 1967, the family planning program was gradually implemented and specifically directed to those women after post-abortion curettage based on providing free access to artificial contraceptive methods such as intrauterine devices (IUDs) and birth control pills [21], and to a lower degree, surgical sterilization for multiparous women with three or more children. Sterilization using pellets of quinacrine was used in a few hospitals from 1970 [23]. In general, the ratio of acceptation for artificial contraception methods among women post clandestine-induced abortion vs. post partum was 3:1 in the first decade, and the IUD was the preferred method [21]. Current rates for artificial contraception in Chile are comparatively lower than in developed nations, reaching 36%, combining the use of hormonal methods and IUDs in the female population of fertile age [24].

Finally, another important historical fact was the legislation that definitively prohibited therapeutic abortion in 1989. Abortion is

considered unconstitutional according to the 1980 Chilean Constitution, which recognizes and protects human life from conception (Article 19, No. 1). Abortion was legal in Chile from 1931 to 1967, with the permission of three physicians or one physician and two witnesses. From 1967 to 1989, abortion was allowed based on the opinion of two physicians. It is documented that an undetermined number of elective abortions were conducted by several medical doctors utilizing the latter law [19,25], but this practice seemed to be strongly restricted after 1973 by the military government [25] until the definitive derogation in 1989. In practical terms, the current law does not prohibit the previable delivery of a fetus to save the life of the mother because it is interpreted as a medical ethics decision [26].

Table S1. International Classification of Diseases (ICD) 7th-10th versions for classifying maternal death causes in Chile.

Group†	Group List A ICD-7	ICD-7‡ (1957-1967)	ICD-8 (1968-1979)	ICD-9 (1980-1996)	ICD-10 (1997-present)
Sepsis	Sepsis of pregnancy, childbirth and the puerperium (A115)	640, 641, 681, 682, 684	670, 671, 673	670, 671, 673	O22, O23, O85 - O88
Hypertension, eclampsia, toxaemias	Toxaemias of pregnancy and the puerperium (A116)	642, 652, 685, 686	636 - 639	642	O10 - O16
Haemorrhage	Haemorrhage of pregnancy and childbirth (A117)	643, 644, 670 – 672	632, 651 – 653	640, 641, 666, 667	O20, O43 - O46, O67, O72, O73
Abortion	Abortion with and without mention of sepsis or toxaemia (A118 and A119)	650, 651	640 – 645	632, 634 - 639	O03 - O08
Other direct and indirect obstetric causes of death (including ectopic pregnancy)	Other complications of pregnancy, childbirth and the puerperium. Delivery without mention of complication (A120)	645 – 649, 660, 673 – 680, 683, 687 – 689	630, 631 633 - 635 650 654 - 662 672 674 - 678	630, 631, 633 643 - 648 650 - 665 668, 669 672 674 - 677	O00 – O02, O21, O24 – O26, O28 – O36, O40 – O42, O47, O48, O60 – O66, O68 – O71, O74, O75, O80 – O84, O89 – O92, O94 – O99

† Group names are based on the content of the ICD-7, list A (Intermediate list of 150 causes for tabulation of morbidity and mortality).
‡ During 1957 the ICD-6 was used in Chile, but maternal causes of death were directly homologated with the ICD-7.

Classification of maternal death causes

On statistical analysis, we used the total number of deaths to represent general maternal mortality and corrected live births per year in the time series. Thus, the MMR per 100,000 live births was directly calculated. Additionally, because one-to-one matching of the ICD codes was not possible, we estimated the relative importance of different causes, grouping similar causes of maternal deaths together and simultaneously trying to respect the original codification. In particular, exact homologation

was impossible because ICD-9 and ICD-10 included unequal codes for several causes not included in ICD-7 and ICD-8. Therefore, the oldest ICD was used as a reference for constructing the different mortality groups (Table S1).

The main groups used from ICD-7 were A115 (sepsis of pregnancy, childbirth and puerperium), A116 (toxemias of pregnancy and puerperium), A117 (haemorrhage of pregnancy and childbirth), A118 and A119 together (abortions with and without mention of sepsis or toxemia), and A120 (other direct and indirect complications of pregnancy, childbirth and puerperium and delivery without mention of complications) (Table S2). We classified the specific causes from ICD-7, ICD-8, ICD-9 and ICD-10 in one of those five groups. Ectopic pregnancy, hydatidiform mole and other abnormal products of conceptions were excluded from the total number of abortion deaths and were consequently included in the group of other direct and indirect causes of maternal mortality.

Regarding the specific topic on abortion, the ICD-7 classified "abortion" separately distinguishing the presence of sepsis and/or toxaemias. Spontaneous abortion was not explicitly defined. Ectopic pregnancy was separately identified. Abortions related with molar pregnancy and other abnormal products of conception characterized by toxaemias without sepsis were separately considered in the code 652. We considered as death by "abortion" the codes 650 and 651 (A118 and A119 in the list of 150 causes). As a consequence, we could not identify spontaneous abortions complicated by sepsis between 1957 and 1967.

The ICD-8 introduces the codes 640 (abortion induced for medical indications) and 641 (abortion induced for other legal indications) classified using the code A114 in the list of 150 causes (Table S3). The code A115 included the codes 642 (abortion induced for other reasons), 643 (spontaneous abortion), 644 (abortion not specified as induced or spontaneous) and 645 (other abortion). The Chilean database did use the codes A114 and A115 for classifying deaths due to "abortion". Ectopic pregnancy was separately identified. As a consequence, we could not separate accurately deaths by spontaneous abortions between 1968 and 1979 in the Chilean registry.

During the period 1980 to 1996, ICD-9 was used (Table S4). Abortion deaths were represented by codes 632 (missed abortion), 634 (spontaneous abortion), 635 (legal induced abortion), 636 (illegal induced abortion), 637 (unspecified abortion), 638 (attempted but failed abortion), and 639 (complications following abortion and ectopic and molar pregnancies). The study did not consider codes 630 (hydatidiform mole), 631 (Other abnormal products of conception) and 633 (ectopic pregnancy), present in the ICD-9, as death caused by abortion, mainly because abortion is not the primary cause of death. Deaths by spontaneous abortion were identified during this period.

The ICD-10 did not use a specific code for "induced abortion" (Table S5). From 1997, deaths due to complications of induced abortions are classified by medical doctors using the code O06 from ICD-10 (unspecified abortion). Spontaneous abortion, ectopic pregnancy, medical abortion, and other abnormal products of conception are classified according the corresponding codes of the ICD-10. In strict rigour, a precise identification of induced abortions can not be definitively established from death registries using the ICD-10 (*e.g.* in some cases a similitude between incomplete spontaneous abortion and incomplete self-induced abortion exist). Nevertheless, when a death is classified using the code O06 by medical doctors, it is suspected that this may be secondary to induced abortion complications. The number of deaths by "unspecified abortion" was two out of 41 maternal deaths in 2008, and one out of 43 maternal deaths in 2009. No deaths by spontaneous abortions have occurred in the last years and deaths by ectopic pregnancy were three in 2008 and two in 2009. Similar to ICD-

9, codes O00 (ectopic pregnancy), O01 (hydatidiform mole) and O02 (Other abnormal products of conception) were considered individually when deaths by these causes were registered (although the incidence of deaths by these problems is very low).

In general terms, we observed few deaths by spontaneous abortion between 1980 and 2007. Nevertheless, because we can not accurately identify deaths by spontaneous abortion during the periods in which the ICD-7 and ICD-8 were utilized and in order to avoid any biased calculation in the proportion of abortion related-deaths we consider "abortion deaths" including spontaneous abortion but excluding ectopic pregnancy and other abnormal products of conception characterized by toxaemias during pregnancy (*e.g.* hydatidiform mole).

Table S2. International Classification of Diseases (ICD) 7th version for classifying maternal death causes in Chile.

Group List A ICD-7	ICD-7‡ (1957-1967)	Causes of death
Sepsis of pregnancy, childbirth and the puerperium (A115)	640	Pyelitis and pyelonephritis of pregnancy
	641	Other infections of genito-urinary tract during pregnancy
	681	Sepsis of childbirth and the puerperium
	682	Puerperal phlebitis and thrombosis
	684	Puerperal pulmonary embolism
Toxaemias of pregnancy and the puerperium (A116)	642	Toxaemias of pregnancy
	652	Abortion with toxaemia, without mention of sepsis
	685	Puerperal eclampsia
	686	Other forms of puerperal toxaemia
Haemorrhage of pregnancy and childbirth (A117)	643	Placenta praevia
	644	Other haemorrhage of pregnancy
	670	Delivery complicated by placenta praevia or antepartum haemorrhage
	671	Delivery complicated by retained placenta
	672	Delivery complicated by other postpartum haemorrhage
Abortion with and without mention of sepsis or toxaemia (A118 and A119)	650	Abortion without mention of sepsis or toxaemia
	651	Abortion with sepsis
Other complications of pregnancy, childbirth and the puerperium. Delivery without mention of complication (A120)	645	Ectopic pregnancy
	646	Anaemia of pregnancy
	647	Pregnancy with malposition of foetus in uterus
	648	Other complications arising from pregnancy
	649	Pregnancy associated with other conditions
	660	Delivery without mention of complication
	673	Delivery complicated by abnormality of bony pelvis
	674	Delivery complicated by disproportion or malposition of foetus
	675	Delivery complicated by prolonged labour of other origin
	676	Delivery with laceration of perineum, without mention of other laceration
	677	Delivery with other trauma
	678	Delivery with other complications of childbirth
	680	Puerperal urinary infection without other sepsis
	683	Pyrexia of unknown origin during the puerperium
	687	Cerebral haemorrhage in the puerperium
	688	Other and unspecified complications of the puerperium
	689	Mastitis and other disorders of lactation

† Group names are based on the content of the ICD-7, list A (Intermediate list of 150 causes for tabulation of morbidity and mortality).
‡ During 1957 the ICD-6 was used in Chile, but maternal causes of death were directly homologated with the ICD-7.

Table S3. International Classification of Diseases (ICD) 8th version for classifying maternal death causes in Chile. Homologation with five groups from ICD 7th version, list A.

Group List A ICD-7†	ICD-8 (1968-1979)	Cause of death
Sepsis of pregnancy, childbirth and the puerperium (A115)	670	Sepsis of childbirth and the puerperium
	671	Puerperal phlebitis and thrombosis
	673	Puerperal pulmonary embolism
Toxaemias of pregnancy and the puerperium (A116)	636	Renal disease arising during pregnancy and the puerperium
	637	Pre-eclampsia, eclampsia and toxaemia, unspecified
	638	Hyperemesis gravidarum
	639	Other toxaemias of pregnancy and the puerperium
Haemorrhage of pregnancy and childbirth (A117)	632	Haemorrhage of pregnancy
	651	Delivery complicated by placenta praevia or antepartum haemorrhage
	652	Delivery complicated by retained placenta
	653	Delivery complicated by other postpartum haemorrhage
Abortion with and without mention of sepsis or toxaemia (A118 and A119)	640	Abortion induced for medical indications
	641	Abortion induced for other legal indications
	642	Abortion induced for other reasons
	643	Spontaneous abortion
	644	Abortion not specified as induced or spontaneous
	645	Other abortion
Other complications of pregnancy, childbirth and the puerperium. Delivery without mention of complication (A120)	630	Infections of genital tract during pregnancy
	631	Ectopic pregnancy
	633	Anaemia of pregnancy
	634	Other complications of pregnancy
	635	Urinary infections arising during pregnancy and the puerperium
	650	Delivery without mention of complication
	654	Delivery complicated by abnormality of bony pelvis
	655	Delivery complicated by foetopelvic disproportion
	656	Delivery complicated by malpresentation of foetus
	657	Delivery complicated by prolonged labour of other origin
	658	Delivery with laceration of perineum, without mention of other laceration
	659	Delivery with rupture of uterus
	660	Delivery with other obstetrical trauma
	661	Delivery with other complications
	662	Anaesthetic death in uncomplicated delivery
	672	Pyrexia of unknown origin during the puerperium
	674	Cerebral haemorrhage in the puerperium
	675	Puerperal blood dyscrasias
	676	Anaemia of puerperium
	677	Other and unspecified complications of the puerperium
	678	Mastitis and other disorders of lactation

† Group names are based on the content of the ICD-7, list A (Intermediate list of 150 causes for tabulation of morbidity and mortality).

Table S4. International Classification of Diseases (ICD) 9th version for classifying maternal death causes in Chile. Homologation with five groups from ICD 7th version, list A.

Group List A ICD-7†	ICD-9 (1980-1996)	Cause of death
Sepsis of pregnancy, childbirth and the puerperium (A115)	670	Major puerperal infection
	671	Venous complications in pregnancy and the puerperium
	673	Obstetrical pulmonary embolism
Toxaemias of pregnancy and the puerperium (A116)	642	Hypertension complicating pregnancy, childbirth and the puerperium
Haemorrhage of pregnancy and childbirth (A117)	640	Haemorrhage in early pregnancy
	641	Antepartum haemorrhage, abruptio placentae and placenta praevia
	666	Postpartum haemorrhage
	667	Retained placenta or membranes, without haemorrhage
Abortion with and without mention of sepsis or toxaemia (A118 and A119)	632	Missed abortion
	634	Spontaneous abortion
	635	Legal induced abortion
	636	Illegal induced abortion
	637	Unspecified abortion
	638	Attempted but failed abortion
	639	Complications following abortion and ectopic and molar pregnancies
Other complications of pregnancy, childbirth and the puerperium. Delivery without mention of complication (A120)	630	Hydatidiform mole
	631	Other abnormal product of conception
	633	Ectopic pregnancy
	643	Excessive vomiting in pregnancy
	644	Early or threatened labour
	645	Prolonged pregnancy
	646	Other complications of pregnancy, not elsewhere classified
	647	Infective and parasitic conditions in the mother classifiable elsewhere but complicating pregnancy, childbirth and the puerperium
	648	Other current conditions in the mother classifiable elsewhere but complicating pregnancy, childbirth and the puerperium
	650	Delivery in a completely normal case
	651	Multiple gestation
	652	Malposition and malpresentation of foetus
	653	Disproportion
	654	Abnormality of organs and soft tissues of pelvis
	655	Known or suspected foetal abnormality affecting management of mother
	656	Other foetal and placental problems affecting management of mother
	657	Polyhydramnios
	658	Other problems associated with amniotic cavity and membranes
	659	Other indications for care or intervention related to labour and delivery and not elsewhere classified
	660	Obstructed labour
	661	Abnormality of forces of labour
	662	Long labour
	663	Umbilical cord complications
	664	Trauma to perineum and vulva during delivery
	665	Other obstetrical trauma
	668	Complications of the administration of anaesthetic or other sedation in labour and delivery
	669	Other complications of labour and delivery, not elsewhere classified
	672	Pyrexia of unknown origin during the puerperium
	674	Other and unspecified complications of the puerperium, not elsewhere classified
	675	Infections of the breast and nipple associated with childbirth
	676	Other disorders of the breast associated with childbirth, and disorders of lactation
	677	Late effect of complication of pregnancy, childbirth and the puerperium

† Group names are based on the content of the ICD-7, list A (Intermediate list of 150 causes for tabulation of morbidity and mortality).

Table S5. International Classification of Diseases (ICD) 10th version for classifying maternal death causes in Chile. Homologation with five groups from ICD 7th version, list A.

Group List A ICD-7†	ICD-10 (1997-present)	Causes of death
Sepsis of pregnancy, childbirth and the puerperium (A115)	O22	Venous complications in pregnancy
	O23	Infections of genitourinary tract in pregnancy.
	O85	Puerperal sepsis
	O86	Other puerperal infections
	O87	Venous complications in the puerperium
	O88	Obstetric embolism
Toxaemias of pregnancy and the puerperium (A116)	O10	Pre-existing hypertension complicating pregnancy, childbirth and the puerperium
	O11	Pre-existing hypertensive disorder with superimposed proteinuria
	O12	Gestational [pregnancy-induced] oedema and proteinuria without hypertension
	O13	Gestational [pregnancy-induced] hypertension without significant proteinuria
	O14	Gestational [pregnancy-induced] hypertension with significant proteinuria
	O15	Eclampsia
	O16	Unspecified maternal hypertension
Haemorrhage of pregnancy and childbirth (A117)	O20	Haemorrhage in early pregnancy
	O43	Placental disorders
	O44	Placenta praevia
	O45	Premature separation of placenta [abruptio placentae]
	O46	Antepartum haemorrhage, not elsewhere classified
	O67	Labour and delivery complicated by intrapartum haemorrhage, not elsewhere classified
	O72	Postpartum haemorrhage
	O73	Retained placenta and membranes, without haemorrhage
Abortion with and without mention of sepsis or toxaemia (A118 and A119)	O03	Spontaneous abortion
	O04	Medical abortion
	O05	Other abortion
	O06	Unspecified abortion
	O07	Failed attempted abortion
	O08	Complications following abortion and ectopic and molar pregnancy
Other complications of pregnancy, childbirth and the puerperium. Delivery without mention of complication (A120)	O00	Ectopic pregnancy
	O01	Hydatidiform mole
	O02	Other abnormal products of conception
	O21	Excessive vomiting in pregnancy
	O24	Diabetes mellitus in pregnancy
	O25	Malnutrition in pregnancy
	O26	Maternal care for other conditions predominantly related to pregnancy
	O28	Abnormal findings on antenatal screening of mother
	O29	Complications of anaesthesia during pregnancy
	O30	Multiple gestation
	O31	Complications specific to multiple gestation
	O32	Maternal care for known or suspected malpresentation of fetus
	O33	Maternal care for known or suspected disproportion
	O34	Maternal care for known or suspected abnormality of pelvic organs
	O35	Maternal care for known or suspected fetal abnormality and damage
	O36	Maternal care for other known or suspected fetal problems
	O40	Polyhydramnios
	O41	Other disorders of amniotic fluid and membranes
	O42	Premature rupture of membranes
	O47	False labour
	O48	Prolonged pregnancy
	O60	Preterm labour and delivery
	O61	Failed induction of labour
	O62	Abnormalities of forces of labour
	O63	Long labour
	O64	Obstructed labour due to malposition and malpresentation of fetus
	O65	Obstructed labour due to maternal pelvic abnormality
	O66	Other obstructed labour
	O68	Labour and delivery complicated by fetal stress [distress]
	O69	Labour and delivery complicated by umbilical cord complications
	O70	Perineal laceration during delivery
	O71	Other obstetric trauma
	O74	Complications of anaesthesia during labour and delivery
	O75	Other complications of labour and delivery, not elsewhere classified

† Group names are based on the content of the ICD-7, list A (Intermediate list of 150 causes for tabulation of morbidity and mortality).

Table S5. International Classification of Diseases (ICD) 10th version for classifying maternal death causes in Chile. Homologation with five groups from ICD 7th version, list A.

(Table S5, continued)

Group List A ICD-7†	ICD-10 (1997-present)	Causes of death
Other complications of pregnancy, childbirth and the puerperium. Delivery without mention of complication (A120)	O80	Single spontaneous delivery
	O81	Single delivery by forceps and vacuum extractor
	O82	Single delivery by caesarean section
	O83	Other assisted single delivery
	O84	Multiple delivery
	O89	Complications of anaesthesia during the puerperium
	O90	Complications of the puerperium, not elsewhere classified
	O91	Infections of breast associated with childbirth
	O92	Other disorders of breast and lactation associated with childbirth
	O94	Sequelae of complication of pregnancy, childbirth and the puerperium
	O95	Obstetric death of unspecified cause
	O96	Death from any obstetric cause occurring more than 42 days but less than one year after delivery
	O97	Death from sequelae of direct obstetric causes
	O98	Maternal infectious and parasitic diseases classifiable elsewhere but complicating pregnancy, childbirth and the puerperium
	O99	Other maternal diseases classifiable elsewhere but complicating pregnancy, childbirth and the puerperium

† Group names are based on the content of the ICD-7, list A (Intermediate list of 150 causes for tabulation of morbidity and mortality).

REFERENCES

1. Report of the International Conference for the Seventh Revision of the International Lists of Diseases and Causes of Death. Geneva, World Health Organization, 1955 (unpublished document WHO/HA/7 Rev. Conf./17 Rev. 1; available on request from Strengthening of Epidemiological and Statistical Services, World Health Organization, 1211 Geneva 27, Switzerland).
2. Report of the International Conference for the Eighth Revision of the International Classification of Diseases. Geneva, World Health Organization, 1965 (unpublished document WHO/ICD9/74.4; available on request from Strengthening of Epidemiological and Statistical Services, World Health Organization, 1211 Geneva 27, Switzerland).
3. Manual of the international statistical classification of diseases, injuries, and causes of death. (1977) Volume 1. Geneva, World Health Organization.
4. International Statistical Classification of Diseases and Related Health Problems, 10th Revision. Geneva, World Health Organization. Available: http://www.who.int/classifications/icd/en/ Accessed: 2012 Apr 13.
5. Instituto Nacional de Estadística. (1999) Anuario de demografía 1997. Available: http://www.ine.cl/canales/chile_estadistico/demografia_y_vitales/estadisticas_vitales/pdf/anuarios/demografia1997.zip. Accessed: 2012 Apr 13.
6. Instituto Nacional de Estadística. (2000) Anuario de demografía 1998. Available: http://www.ine.cl/canales/chile_estadistico/demografia_y_vitales/estadisticas_vitales/pdf/anuarios/demografia1998.zip. Accessed: 2012 Apr 13.
7. Instituto Nacional de Estadística. (2009) Estadísticas Vitales, Informe Anual 2007. Available: http://www.ine.cl/canales/chile_estadistico/demografia_y_vitales/estadisticas_vitales/2010/04_01_10/vitales2007.pdf. Accessed: 2012 Apr 13.
8. Banco Central de Chile (2001) Indicadores económicos y sociales de Chile 1960-2000. Santiago de Chile: Departamento de Publicaciones, Banco Central de Chile. 834 p.
9. Braun-Llona J, Braun-Llona M, Briones I, Díaz J, Lüders R (1998) Economía Chilena 1810-1995. Estadísticas Históricas. PUC Economics Institute Working Paper No. 187. Available: http://www.economia.puc.cl/docs/dt_187.pdf. Accessed: 2012 Apr 13.

10. Fridley BL, McDonnell SK, Rabe KG, Tang R, Biernacka JM, et al. (2009) Single versus multiple imputation for genotypic data. *BMC Proc* 3 Suppl 7:S7
11. Honaker J, King G. (2010) What to do about missing values in time-series cross-section data. *Am J Polit Sci* 54: 561–581.
12. Maddison A. (2010) Historical Statistical of the World Economy: 1-2008 AD. Available: http://www.ggdc.net/maddison/Historical_Statistics/horizontal-file_02-2010.xls. Accessed: 2012 Apr 13.
13. Superintendecia de Servicios Sanitarios. (2010) Evolución Histórica Cobertura Nacional Agua Potable y Alcantarillado Urbanos desde 1965. Available: http://www.siss.gob.cl/article-3683.html. Accessed 2012 Apr 13.
14. Valenzuela S, Jouravlev A. (2007) Servicios de agua potable y alcantarillado en Chile: factores determinantes del desempeño. Naciones Unidas. Available: http://www.eclac.cl/publicaciones/xml/0/28650/lcl2727e.pdf. Accessed: 2012 Apr 13.
15. Castañeda T (1985) The determinants of the decline in infant mortality in Chile, 1975-1982. *Cuad Econ* 22: 195-214.
16. Huneeus C, Paz Lanas M (2002) Political science and history: Eduardo Cruz-Coke and the welfare state in Chile, 1937-38. *Historia Santiago* 35: 151-186.
17. Monckeberg F, Valiente S, Mardones F (1987) Infant and pre-school nutrition: Economical development, versus intervention strategies; The case of Chile. *Nutr Res* 7: 327-342.
18. Monckeberg F, Chichester CO (1978) Chilean experience with fortified children's formulas. *Adv Exp Med Biol* 105: 11-28.
19. Armijo R, Monreal T (1965) The problem of induced abortion in Chile. *Milbank Mem Fund Q* 43: Suppl:263-280.
20. Plaza S, Briones H (1963) Abortion as a social welfare problem. *Rev Med Chile* 32:294-7.
21. Viel B (1969) Results of a family planning program in the western area of the city of Santiago. *Am J Public Health* 59:1898-1909.
22. Szot M J, Moreno W C (2003) Mortalidad por aborto en Chile: análisis epidemiológico 1985-2000. *Rev Chil Obstet Ginecol* 68: 309-314.
23. Zipper J, Trujillo V (2003) 25 years of quinacrine sterilization experience in Chile: review of 2,592 cases. *Int J Gynaecol Obstet* 83 Suppl 2:S23-9.
24. Ministerio de Salud. Gobierno de Chile (2007) Segunda Encuesta Nacional de Calidad de Vida 2006. Informe de resultados total nacional. Available: http://epi.minsal.cl/epi/html/sdesalud/calidaddevida2006/Informe%20Final%20Encuesta%20de%20Calidad%20de%20Vida%20y%20Salud%202006.pdf. Accessed: 2012 Apr 13.
25. Montebruno P, Delgado A (2003) La vía chilena hacia el aborto. The Clinic 2003. Available: http://www.theclinic.cl/2012/03/13/la-via-chilena/. Accessed: 2012 Apr 13.
26. Valenzuela CY (2003) Scientific ethics of therapeutic abortion. *Rev Med Chil* 131:562-8.
