# Supplementary material for: Women's Education Level, Maternal Health Facilities, Abortion Legislation and Maternal Deaths: A Natural Experiment in Chile from 1957 to 2007
Source: PLoS One. 2012 May 4;7(5):e36613. doi: 10.1371/journal.pone.0036613 (PMC3344918; doi:10.1371/journal.pone.0036613)
Supplement: Appendix S2 — Complementary results. (PDF) [file pone.0036613.s002.pdf]

APPENDIX S2
COMPLEMENTARY RESULTS

Table S6. Time series of maternal deaths and selected indicators, Chile 1957 – 2007.

Year	Population at Fertile Age	Live Births	Maternal Deaths	MM Ratio†	MM Rate††
1957	1,646,472	271,905	736	270.7	44.7
1958	1,687,468	273,793	769	280.9	45.6
1959	1,728,465	276,686	672	242.9	38.9
1960	1,768,652	287,063	789	274.9	44.6
1961	1,815,443	296,230	870	293.7	47.9
1962	1,862,235	303,249	787	259.5	42.3
1963	1,909,027	306,575	733	239.1	38.4
1964	1,955,818	303,219	721	237.8	36.9
1965	1,967,861	302,401	787	260.3	40.0
1966	2,010,318	294,438	676	229.6	33.6
1967	2,052,776	273,399	630	230.4	30.7
1968	2,095,233	262,036	550	209.9	26.3
1969	2,137,691	252,157	454	180.1	21.2
1970	2,219,757	251,231	451	179.5	20.3
1971	2,259,149	261,021	335	128.3	14.8
1972	2,298,543	269,576	306	113.5	13.3
1973	2,337,936	291,277	331	113.6	14.2
1974	2,377,329	278,165	313	112.5	13.2
1975	2,548,645	266,513	306	114.8	12.0
1976	2,585,820	256,641	253	98.6	9.8
1977	2,622,995	242,847	214	88.1	8.2
1978	2,660,171	245,763	170	69.2	6.4
1979	2,697,346	251,745	155	61.6	5.8
1980	2,911,821	262,030	135	51.5	4.6
1981	2,959,928	273,882	116	42.4	3.9
1982	3,008,035	278,000	144	51.8	4.8
1983	3,056,142	259,888	107	41.2	3.5
1984	3,104,249	262,317	94	35.8	3.0
1985	3,240,601	263,039	132	50.2	4.1
1986	3,297,507	272,941	129	47.3	3.9
1987	3,354,413	279,367	135	48.3	4.0
1988	3,411,319	297,823	123	41.3	3.6
1989	3,468,226	306,080	125	40.8	3.6
1990	3,552,327	309,220	123	39.8	3.5
1991	3,605,504	300,740	106	35.3	2.9
1992	3,658,683	294,218	91	30.9	2.5
1993	3,711,862	289,419	100	34.6	2.7
1994	3,765,042	285,228	73	25.6	1.9
1995	3,818,221	275,760	86	31.2	2.3
1996	3,874,803	272,163	63	23.2	1.7
1997	3,931,387	265,493	61	23.0	1.6
1998	3,987,971	261,802	55	21.0	1.4
1999	4,044,553	254,096	60	23.6	1.5
2000	4,101,137	252,155	49	19.4	1.2
2001	4,156,091	248,651	45	18.1	1.1
2002	4,211,046	241,027	42	17.4	1.0
2003	4,265,999	236,223	30	12.7	0.7
2004	4,320,954	232,588	42	18.1	1.0
2005	4,375,908	232,092	48	20.7	1.1
2006	4,411,544	233,104	47	20.2	1.1
2007	4,447,180	242,054	44	18.2	1.0

† Referred to maternal mortality ratio, number of deaths per 100,000 live births

† Referred to maternal mortality rate, number of deaths per 100,000 women of fertile age

Segmented regression

To assess the effects of historical periods, a segmented regression technique [1-3] was used to incorporate join points and their correspondent segments into multiple ARIMA models: year 1965, representing a proxy for the change in mandatory schooling legislation and the implementation of the maternal health program and the contraceptive program; and year 1989, representing the start of the legislation that prohibited therapeutic abortion.

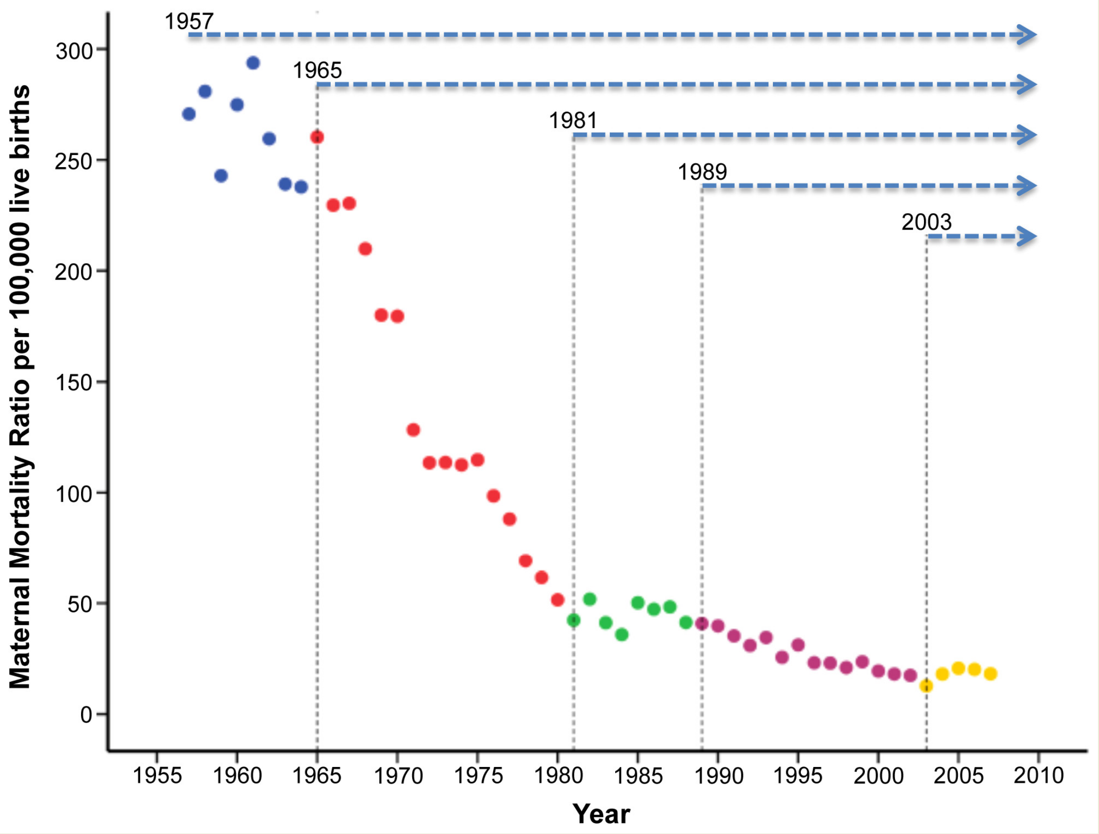

Figure S3. Scatterplot identifying possible join points on maternal mortality trend in Chile for segmented regression analyses.

In the interpolation of the observed curve (Figure S3), in addition to 1989, other cut-off points were selected at 1981 and 2003; that at these points, there was an apparent disruption of the trend. From 1965, we observed a continuous downward trend until 1981, with a visual change in the slope from this point until 2003. The proposed model is as showed on Figure S4.

The terms x_i represent independent variables. In other words, x_i represents selected cut-off points or join points and the segments (time periods) in the time series for each β_i coefficient, respectively.

In the segmented regression ARIMA model utilized in this study, each time segment was additively incorporated following a prospective sequence by year (see Figures S3 and S4). Thus it was assessed if a significant “break” did occur sequentially in the slope of the MMR trend with respect to the join point being evaluated after the previous trend. In consequence, this kind of statistical analysis is unidirectional, that is, prospectively conducted over the entire trend of mortality. Considering the trend from 1957, the first joint point that significantly broken that trend was identified in 1965. Considering the trend from 1965, the second joint point that significantly broken that trend was identified in 1981. Thus, the first and second join points identified to provoke a

significant change in the slope of the MMR were in 1965 and 1981. After 1981, there were no significant changes on the decreasing trends. The joint point breaks of 1989 and 2003 were not statistically significant and, because the slopes for periods 1981-2007 and 1989-2007 were parallel, beta coefficients were not statistically significant (Figure 5).

$$MMR = \beta_0 + \beta_1 x_1 + \beta_2 x_2 + \beta_3 x_3 + \ldots + \beta_i x_i$$

β_0 = constant or average initial value of the MMR from 1957.
β_1 = trend of the curve before the first cut-off point in 1965.
β_2 = immediate change at the 1965 cut-off.
β_3 = trend of the 1965-2007 segment.
β_4 = immediate change at the 1981 cut-off.
β_5 = trend of the 1981-2007 segment.
β_6 = immediate change after the prohibition of abortion in 1989.
β_7 = trend of the 1989-2007 segment.
β_8 = year 2003, when the curve stops descending.
β_9 = stagnant period between 2003 and 2007.

Figure S4. Proposed segmented regression model applied to the Chilean time series from 1957 to 2007.

Other alternative join points such as 1967, 1971, 1975, 1979 and 1985 were tested in alternative regression models, but the results were similar to previous ones. Therefore, these alternative points were ruled out.

World Health Organization (WHO) estimates of MMR compared with official domestic data

Table S7. Direct comparison of the MMR estimates by WHO report [4] with available official domestic data in eight countries of the American continent in 2008.

Country	Live births	Maternal deaths	MMR directly calculated†	MMR by WHO report	Difference††	Overestimation (%)
Canada [5,6]	377,886	34	9.0	12	3	33.3
Chile [7]	248,366	41	16.5	26	9.5	57.6
United States [8,9]	4,247,694	795	18.7	24	5.3	28.3
Costa Rica [10]	75,187	25	33.3	44	10.7	32.1
Cuba [11]	122,569	57	46.5	53	6.5	14
Argentina [12]	746,460	296	39.7	70	30.3	76.3
Mexico [13,14]	1,955,284	1,119	57.2	85	27.8	48.6
Colombia [15,16]	715,453	449	62.8	85	22.2	35.4

MMR refers to the maternal mortality ratio per 100,000 live births
†MMR = (Maternal deaths / Live births) x 100,000
†† Calculated as the difference between the MMR directly calculated and the MMR estimated by WHO.

Abortion hospitalizations

The record of hospitalizations from abortion is routine in Chile at least since 1937 [17]. From 1950 the Ministry of Health elaborates and publishes a yearbook with all the diagnosis of hospital discharges, including obstetric outcomes. Table S8 shows the official number of hospitalizations by abortion (whether spontaneous or induced) between 2001 and 2008 and the ratio between observed live births and abortion hospitalizations. This ratio has remained approximately in 7:1.

Table S8. Abortion hospitalizations in Chile based on official data and estimated proportions for clinical spontaneous abortions and clandestine induced abortions for the period 1998-2008.

Year	Observed Live Births†	Abortion hospitalizations [a]	Ratio	Expected clinical spontaneous abortions [b]	Excess in abortion hospitalizations‡	Percent (%) [c]
2001	248,651	34,479	7,1	29,238	5,241	15,2
2002	241,027	34,968	6,8	28,380	6,588	18,8
2003	236,223	33,490	7,0	27,856	5,634	16,8
2004	232,588	33,835	6,8	27,365	6,470	19,1
2005	232,092	33,184	7,0	27,422	5,762	17,4
2006	233,104	33,145	7,0	27,488	5,657	17,1
2007	242,054	32,532	7,4	28,579	3,953	12,2
2008	246,581	33,428	7,4	29,293	4,135	12,4

†Based on data of observed live births (uncorrected) by the Chilean National Institute of Statistics, INE [7]. [(a)] Official number published by the Ministry of Health. Ratio refers to live births/abortions hospitalizations ratio. [(b)] Estimated applying the probabilities of the study by Wang *et al*. [18]. ‡Estimated as the difference between observed [(a)] and expected [(b)] hospitalizations from abortion. [(c)] Estimated proportion of hospitalizations possibly related to induced abortions

The number of expected clinical spontaneous abortions has been estimated utilizing the biological probabilities reported by Wilcox *et al.* [19] and recently corroborated in epidemiological studies by Wang *et al.* [18] It is of note that the biological probabilities of viable conceptions, pregnancies terminated in live births, early pregnancy loss and clinical spontaneous miscarriages are in a dependent relationship with each other and they tend to be relatively stable across different populations [20]. In the study by Wang *et al.* live births presented a probability of 0.665 of all conceptions and clinical spontaneous abortions represented 0.079 [18]. After applying these probabilities, if the observed number of abortion hospitalizations is higher than the expected number of clinical spontaneous abortion hospitalizations we can suspect that this "residual excess" may be related to complications by induced abortions. Using this method, it was estimated that induced abortions might have represented between 12% and 19% of all hospitalizations by abortion in Chile between 2001 and 2008 [21].

REFERENCES

1. Gillings D, Makuc D, Siegel E (1981) Analysis of interrupted time series mortality trends: an example to evaluate regionalized perinatal care. Am J Public Health 71: 38-46.
2. Smith DH, Perrin N, Feldstein A, Yang X, Kuang D, et al. (2006) The impact of prescribing safety alerts for elderly persons in an electronic medical record: an interrupted time series evaluation. Arch Intern Med 166: 1098-1104.

3. Feldstein AC, Smith DH, Perrin N, Yang X, Simon SR, et al. (2006) Reducing warfarin medication interactions: an interrupted time series evaluation. Arch Intern Med 166: 1009-1015.
4. World Health Organization (2010) Trends in maternal mortality: 1990 to 2008. Available: http://whqlibdoc.who.int/publications/2010/9789241500265_eng.pdf. Accessed: 2012 Apr 13.
5. Statistics Canada (STATCAN) Table 102-0535. Deaths by cause, Chapter XV. Available: http://www5.statcan.gc.ca/cansim/pick-choisir?lang=eng&p2=33&id=1020535. Accessed: 2012 Apr 13.
6. Statistics Canada (STATCAN) Table 102-4501. Live births. Available: http://www5.statcan.gc.ca/cansim/pick-choisir?lang=eng&p2=33&id=1024501. Accessed: 2012 Apr 13.
7. Instituto Nacional de Estadística (INE) (2010) Estadísticas Vitales, Informe Anual 2008. Instituto Nacional de Estadística. Available: http://www.ine.cl/canales/menu/publicaciones/calendario_de_publicaciones/pdf/21_12_10/vit_08211210.pdf. Accessed: 2012 Apr 13.
8. Martin J, Hamilton B, Sutton P, Ventura S, Mathews T, et al. (2010) National Vital Statistics Reports. Births: Final Data for 2008. Centers for Disease Control and Prevention. Available: http://www.cdc.gov/nchs/data/nvsr/nvsr59/nvsr59_01.pdf. Accessed: 2012 Apr 13.
9. Miniño A, Murphy S, Xu J, Kochanek K (2011) National Vital Statistics Reports. Deaths: Final Data for 2008. Centers for Disease Control and Prevention. Available: http://www.cdc.gov/nchs/data/nvsr/nvsr59/nvsr59_10.pdf. Accessed: 2012 Apr 13.
10. Instituto Nacional de Estadística y Censo (INEC) Estadísticas vitales 2008. Available: http://www.inec.go.cr/A/MT/Población%20y%20Demograf%C3%ADa/Defunciones/Generales/Publicaciones/C0/2008/Publicaciones%20de%20Estad%C3%ADsticas%20Vitales.pdf. Accessed: 2012 Apr 13.
11. Oficina Nacional de Estadísticas (ONE) Anuario Estadistico de Cuba 2010. Maternal mortality rate and its causes. Available: http://www.one.cu/aec2010/datos/19.20.xls. Accessed: 2012 Apr 13.
12. Dirección de Estadísticas e Información de Salud (DEIS) Estadisticas Vitales. Información Básica 2008. Available: http://www.bvs.org.ar/pdf/anuario08.pdf. Accessed: 2012 Apr 13.
13. Instituto Nacional de Estadística y Geografía (INEGI) Consulta interactiva de datos. Estadísticas de natalidad. Available: http://www.inegi.org.mx/sistemas/olap/proyectos/bd/consulta.asp?p=11092&c=11109&s=est&cl=4#. Accessed: 2012 Apr 13.
14. Sistema Nacional de Información en Salud (SINAIS) Razón de mortalidad materna y defunciones por año de registro, 2002-2008. Available: http://sinais.salud.gob.mx/descargas/xls/RazonMuerteMaternayDefunciones.xls. Accessed: 2012 Apr 13.
15. Departamento Administrativo Nacional de Estadística (DANE) Estadísticas vitales. Defunciones no fetales año 2008. Cuadro 8. Available: http://www.dane.gov.co/files/investigaciones/poblacion/defunciones/defun_2008/cuadro8.xls. Accessed: 2012 Apr 13.
16. Departamento Administrativo Nacional de Estadística (DANE) Estadísticas vitales. Nacimientos año 2008. Cuadro 1. Available: http://www.dane.gov.co/files/investigaciones/poblacion/nacimientos/nac_08/Cuadro1.xls. Accessed: 2012 Apr 13.
17. Armijo R, Requena M (1968) Epidemiologic aspects of abortion in Chile. Public Health Rep 83: 41-48.

18. Wang X, Chen C, Wang L, Chen D, Guang W, et al. (2003) Conception, early pregnancy loss, and time to clinical pregnancy: a population-based prospective study. Fertil Steril 79: 577-584.
19. Wilcox AJ, Weinberg CR, Baird DD (1995) Timing of sexual intercourse in relation to ovulation. Effects on the probability of conception, survival of the pregnancy, and sex of the baby. N Engl J Med 333: 1517-1521.
20. Lynch C, Jackson L, Buck Louis G (2006) Estimation of the day-specific probabilities of conception: current state of the knowledge and the relevance for epidemiological research. Paediatr Perinat Epidemiol 20: 3-12.
21. Koch E, Bravo M, Gatica S, Stecher J, Aracena P, et al. (2012) Abortion ovestimated in Latin America: a critical review. Ginecol Obstet Mex: in press.
