# Supplementary figures and images for: Women's Education Level, Maternal Health Facilities, Abortion Legislation and Maternal Deaths: A Natural Experiment in Chile from 1957 to 2007
Source: PLoS One. 2012 May 4;7(5):e36613. doi: 10.1371/journal.pone.0036613 (PMC3344918; doi:10.1371/journal.pone.0036613)

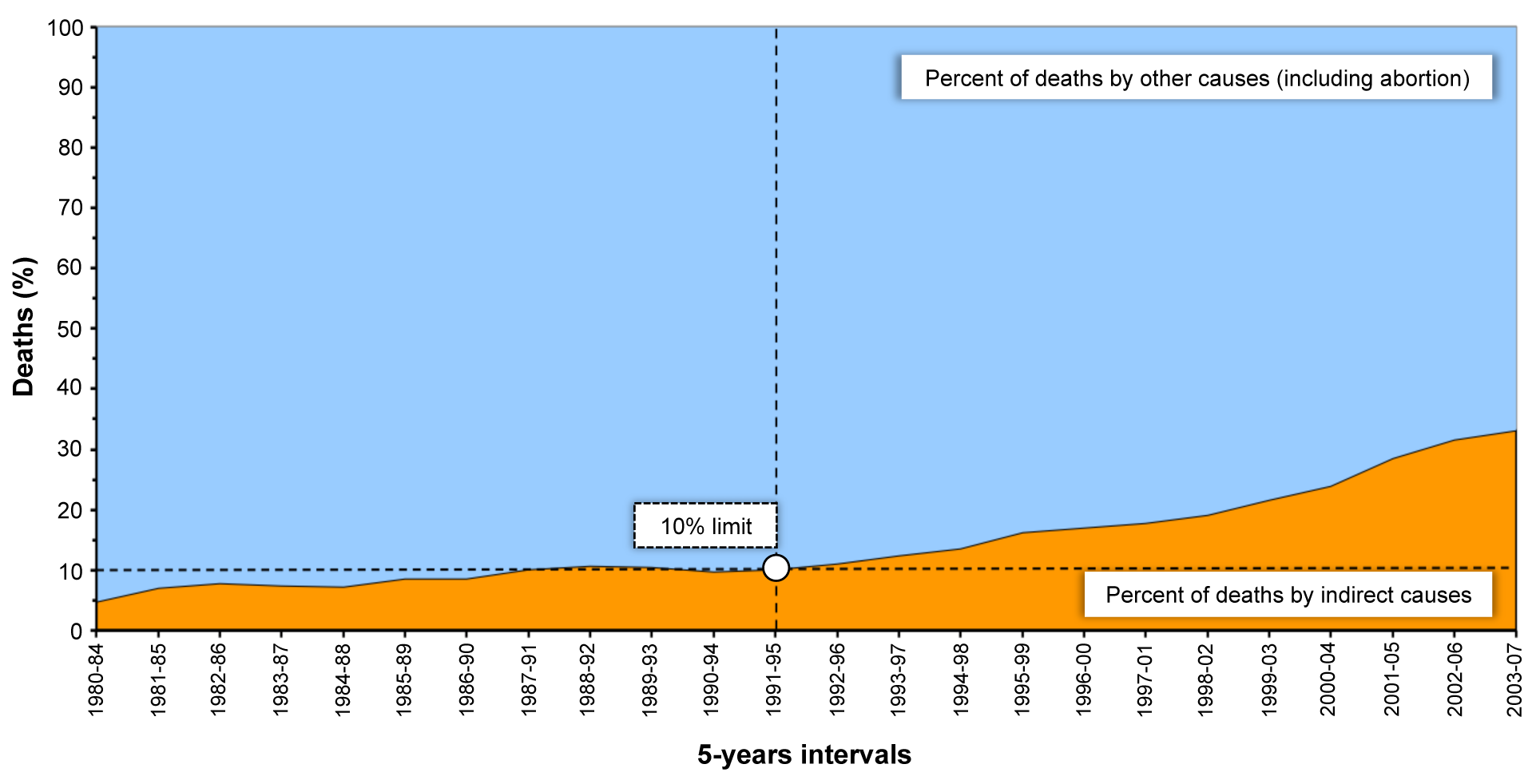

Supplement: Figure S1 — Relative importance of maternal deaths by indirect causes (percent from all maternal deaths including abortion) based on 24 continuous five-year intervals between 1980 and 2007 observed in Chile. The intersection (white circle) of the broken-lines identifies the interval 1991–1995 as the point in which the percent of indirect causes of maternal death began to progressively increase beyond the limit of 10%. During the period of analysis the International Classification of Diseases codes version 9th (1980 to 1996) and 10th were used (1997 to present). (TIF) [file pone.0036613.s003.tif]

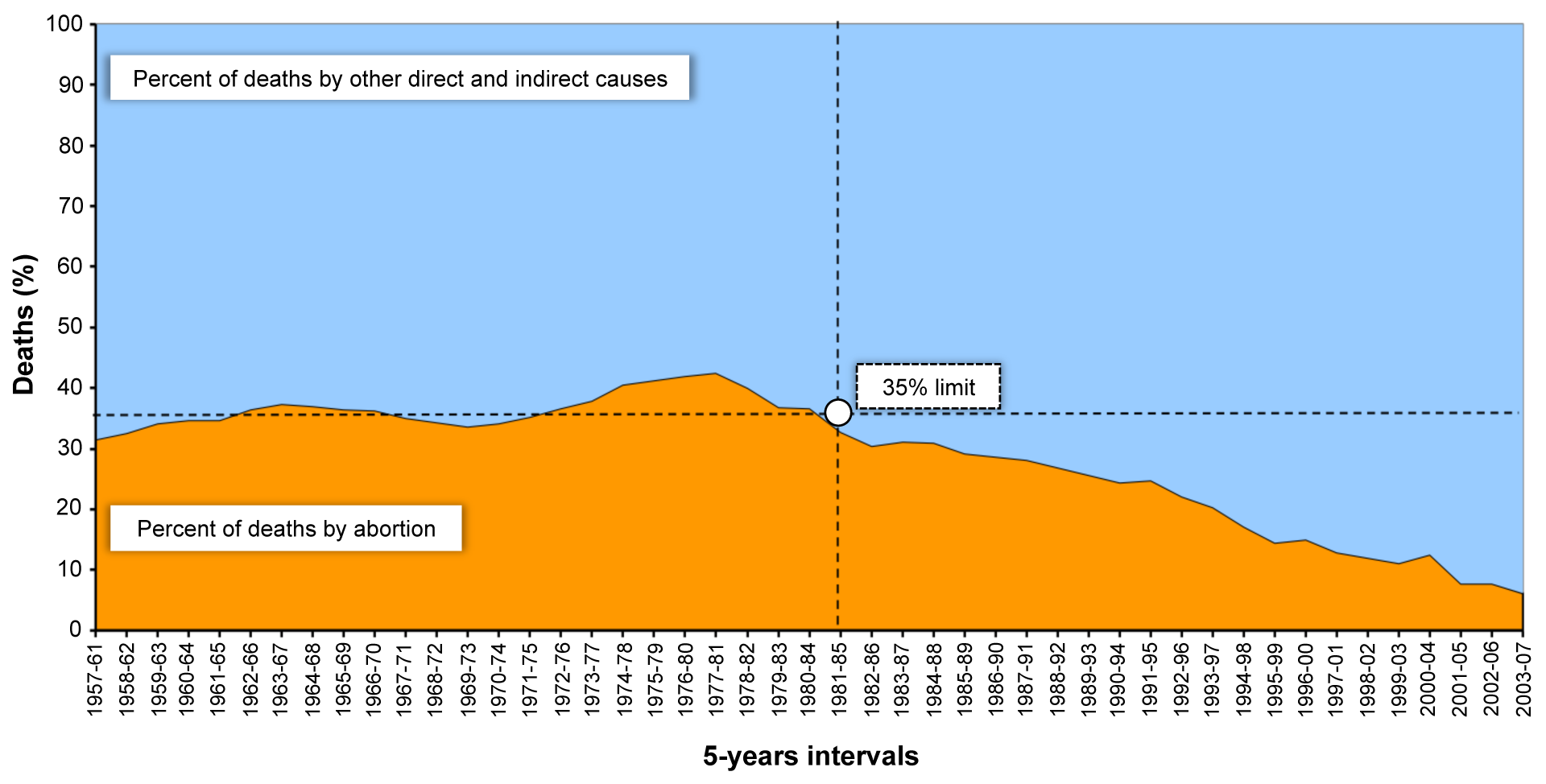

Supplement: Figure S2 — Relative importance of death by abortion (percent from all direct and indirect maternal causes of death) based on 47 continuous five-year intervals between 1957 and 2007 observed in Chile. The intersection (white circle) of the broken-lines identifies the interval 1981–1985 as the point in which the percent of abortion deaths began to progressively decrease under the limit of 35%. During the period of analysis the International Classification of Diseases codes version 7th (1957 to 1967), 8th (1968 to 1979), 9th (1980 to 1996) and 10th were used (1997 to present). The percents were calculated considering all deaths by spontaneous and induced abortions combined but excluding ectopic pregnancy, hydatidiform mole and other abnormal products of conception. (TIF) [file pone.0036613.s004.tif]

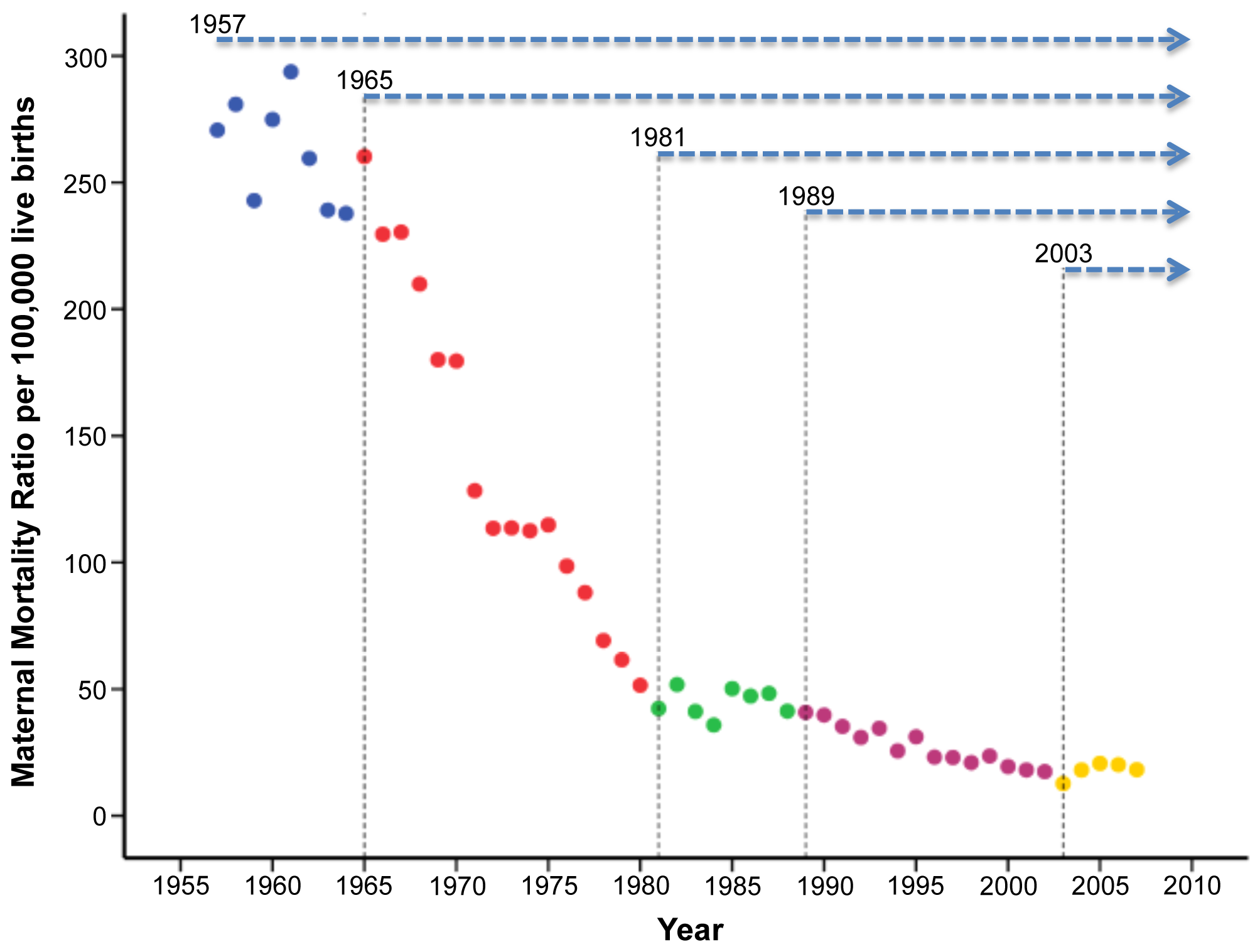

Supplement: Figure S3 — Scatterplot identifying possible join points on maternal mortality trend in Chile for segmented regression analyses. (TIF) [file pone.0036613.s005.tif]

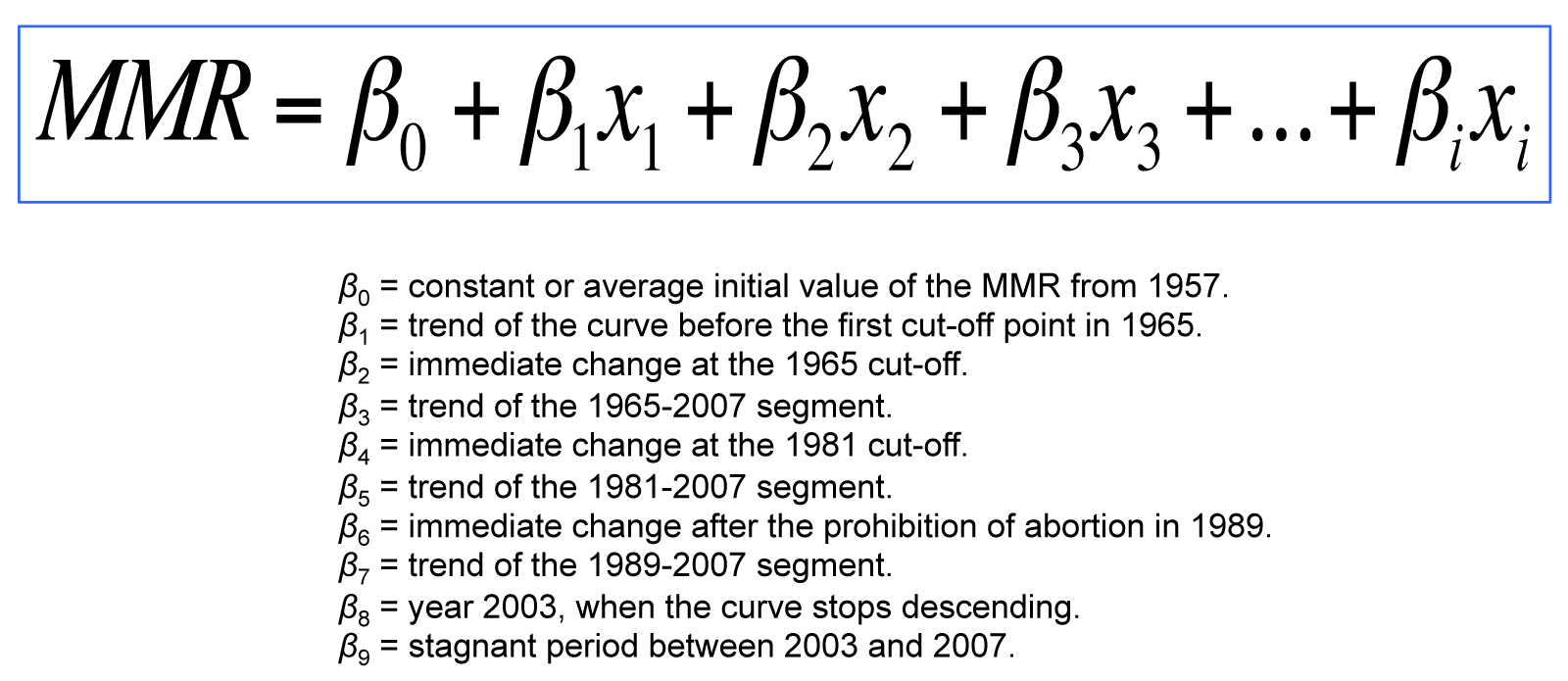

Supplement: Figure S4 — Proposed segmented regression model applied to the Chilean time series from 1957 to 2007. (TIF) [file pone.0036613.s006.tif]
