# Supplementary material for: Women's Education Level, Maternal Health Facilities, Abortion Legislation and Maternal Deaths: A Natural Experiment in Chile from 1957 to 2007
Source: PLoS One. 2012 May 4;7(5):e36613. doi: 10.1371/journal.pone.0036613 (PMC3344918; doi:10.1371/journal.pone.0036613)
Supplement: Table S1 — International Classification of Diseases (ICD) 7th–10th versions for classifying maternal death causes in Chile. The groups for homologation were selected from ICD 7th version, list A. (PDF) [file pone.0036613.s007.pdf]

Table S1. International Classification of Diseases (ICD) 7th-10th versions for classifying maternal death causes in Chile. The groups for homologation were selected from ICD 7th version, list A.

Group†	Group List A ICD-7	ICD-7‡ (1957-1967)	ICD-8 (1968-1979)	ICD-9 (1980-1996)	ICD-10 (1997-present)
Sepsis	Sepsis of pregnancy, childbirth and the puerperium (A115)	640, 641, 681, 682, 684	670, 671, 673	670, 671, 673	O22, O23, O85 - O88
Hypertension, eclampsia, toxaemias	Toxaemias of pregnancy and the puerperium (A116)	642, 652, 685, 686	636 - 639	642	O10 - O16
Haemorrhage	Haemorrhage of pregnancy and childbirth (A117)	643, 644, 670 – 672	632, 651 – 653	640, 641, 666, 667	O20, O43 - O46, O67, O72, O73
Abortion	Abortion with and without mention of sepsis or toxaemia (A118 and A119)	650, 651	640 – 645	632, 634 - 639	O03 - O08
Other direct and indirect obstetric causes of death (including ectopic pregnancy)	Other complications of pregnancy, childbirth and the puerperium. Delivery without mention of complication (A120)	645 – 649, 660, 673 – 680, 683, 687 – 689	630, 631 633 - 635 650 654 - 662 672 674 - 678	630, 631, 633 643 - 648 650 - 665 668, 669 672 674 - 677	O00 – O02, O21, O24 – O26, O28 – O36, O40 – O42, O47, O48, O60 – O66, O68 – O71, O74, O75, O80 – O84, O89 – O92, O94 – O99

† Group names are based on the content of the ICD-7, list A (Intermediate list of 150 causes for tabulation of morbidity and mortality).
‡ During 1957 the ICD-6 was used in Chile, but maternal causes of death were directly homologated with the ICD-7.
