# Supplementary material for: Women's Education Level, Maternal Health Facilities, Abortion Legislation and Maternal Deaths: A Natural Experiment in Chile from 1957 to 2007
Source: PLoS One. 2012 May 4;7(5):e36613. doi: 10.1371/journal.pone.0036613 (PMC3344918; doi:10.1371/journal.pone.0036613)
Supplement: Table S2 — International Classification of Diseases (ICD) 7th version for classifying maternal death causes in Chile. (PDF) [file pone.0036613.s008.pdf]

Table S2. International Classification of Diseases (ICD) 7th version for classifying maternal death causes in Chile.

Group List A ICD-7	ICD-7[‡] (1957-1967)	Causes of death
Sepsis of pregnancy, childbirth and the puerperium (A115)	640	Pyelitis and pyelonephritis of pregnancy
	641	Other infections of genito-urinary tract during pregnancy
	681	Sepsis of childbirth and the puerperium
	682	Puerperal phlebitis and thrombosis
	684	Puerperal pulmonary embolism
Toxaemias of pregnancy and the puerperium (A116)	642	Toxaemias of pregnancy
	652	Abortion with toxaemia, without mention of sepsis
	685	Puerperal eclampsia
	686	Other forms of puerperal toxaemia
Haemorrhage of pregnancy and childbirth (A117)	643	Placenta praevia
	644	Other haemorrhage of pregnancy
	670	Delivery complicated by placenta praevia or antepartum haemorrhage
	671	Delivery complicated by retained placenta
	672	Delivery complicated by other postpartum haemorrhage
Abortion with and without mention of sepsis or toxaemia (A118 and A119)	650	Abortion without mention of sepsis or toxaemia
	651	Abortion with sepsis
Other complications of pregnancy, childbirth and the puerperium. Delivery without mention of complication (A120)	645	Ectopic pregnancy
	646	Anaemia of pregnancy
	647	Pregnancy with malposition of foetus in uterus
	648	Other complications arising from pregnancy
	649	Pregnancy associated with other conditions
	660	Delivery without mention of complication
	673	Delivery complicated by abnormality of bony pelvis
	674	Delivery complicated by disproportion or malposition of foetus
	675	Delivery complicated by prolonged labour of other origin
	676	Delivery with laceration of perineum, without mention of other laceration
	677	Delivery with other trauma
	678	Delivery with other complications of childbirth
	680	Puerperal urinary infection without other sepsis
	683	Pyrexia of unknown origin during the puerperium
	687	Cerebral haemorrhage in the puerperium
	688	Other and unspecified complications of the puerperium
	689	Mastitis and other disorders of lactation

† Group names are based on the content of the ICD-7, list A (Intermediate list of 150 causes for tabulation of morbidity and mortality).

‡ During 1957 the ICD-6 was used in Chile, but maternal causes of death were directly homologated with the ICD-7.
