# Supplementary material for: Women's Education Level, Maternal Health Facilities, Abortion Legislation and Maternal Deaths: A Natural Experiment in Chile from 1957 to 2007
Source: PLoS One. 2012 May 4;7(5):e36613. doi: 10.1371/journal.pone.0036613 (PMC3344918; doi:10.1371/journal.pone.0036613)
Supplement: Table S3 — International Classification of Diseases (ICD) 8th version for classifying maternal death causes in Chile. Homologation with five groups from ICD 7th version, list A. (PDF) [file pone.0036613.s009.pdf]

Table S3. International Classification of Diseases (ICD) 8th version for classifying maternal death causes in Chile. Homologation with five groups from ICD 7th version, list A.

Group List A ICD-7†	ICD-8 (1968-1979)	Cause of death
Sepsis of pregnancy, childbirth and the puerperium (A115)	670	Sepsis of childbirth and the puerperium
	671	Puerperal phlebitis and thrombosis
	673	Puerperal pulmonary embolism
Toxaemias of pregnancy and the puerperium (A116)	636	Renal disease arising during pregnancy and the puerperium
	637	Pre-eclampsia, eclampsia and toxaemia, unspecified
	638	Hyperemesis gravidarum
	639	Other toxaemias of pregnancy and the puerperium
Haemorrhage of pregnancy and childbirth (A117)	632	Haemorrhage of pregnancy
	651	Delivery complicated by placenta praevia or antepartum haemorrhage
	652	Delivery complicated by retained placenta
	653	Delivery complicated by other postpartum haemorrhage
Abortion with and without mention of sepsis or toxaemia (A118 and A119)	640	Abortion induced for medical indications
	641	Abortion induced for other legal indications
	642	Abortion induced for other reasons
	643	Spontaneous abortion
	644	Abortion not specified as induced or spontaneous
	645	Other abortion
Other complications of pregnancy, childbirth and the puerperium. Delivery without mention of complication (A120)	630	Infections of genital tract during pregnancy
	631	Ectopic pregnancy
	633	Anaemia of pregnancy
	634	Other complications of pregnancy
	635	Urinary infections arising during pregnancy and the puerperium
	650	Delivery without mention of complication
	654	Delivery complicated by abnormality of bony pelvis
	655	Delivery complicated by foetopelvic disproportion
	656	Delivery complicated by malpresentation of foetus
	657	Delivery complicated by prolonged labour of other origin
	658	Delivery with laceration of perineum, without mention of other laceration
	659	Delivery with rupture of uterus
	660	Delivery with other obstetrical trauma
	661	Delivery with other complications
	662	Anaesthetic death in uncomplicated delivery
	672	Pyrexia of unknown origin during the puerperium
	674	Cerebral haemorrhage in the puerperium
	675	Puerperal blood dyscrasias
	676	Anaemia of puerperium
	677	Other and unspecified complications of the puerperium
	678	Mastitis and other disorders of lactation

† Group names are based on the content of the ICD-7, list A (Intermediate list of 150 causes for tabulation of morbidity and mortality).
