# Supplementary material for: Women's Education Level, Maternal Health Facilities, Abortion Legislation and Maternal Deaths: A Natural Experiment in Chile from 1957 to 2007
Source: PLoS One. 2012 May 4;7(5):e36613. doi: 10.1371/journal.pone.0036613 (PMC3344918; doi:10.1371/journal.pone.0036613)
Supplement: Table S4 — International Classification of Diseases (ICD) 9th version for classifying maternal death causes in Chile. Homologation with five groups from ICD 7th version, list A. (PDF) [file pone.0036613.s010.pdf]

Table S4. International Classification of Diseases (ICD) 9th version for classifying maternal death causes in Chile. Homologation with five groups from ICD 7th version, list A.

Group List A ICD-7†	ICD-9 (1980-1996)	Cause of death
Sepsis of pregnancy, childbirth and the puerperium (A115)	670	Major puerperal infection
	671	Venous complications in pregnancy and the puerperium
	673	Obstetrical pulmonary embolism
Toxaemias of pregnancy and the puerperium (A116)	642	Hypertension complicating pregnancy, childbirth and the puerperium
Haemorrhage of pregnancy and childbirth (A117)	640	Haemorrhage in early pregnancy
	641	Antepartum haemorrhage, abruptio placentae and placenta praevia
	666	Postpartum haemorrhage
	667	Retained placenta or membranes, without haemorrhage
Abortion with and without mention of sepsis or toxaemia (A118 and A119)	632	Missed abortion
	634	Spontaneous abortion
	635	Legal induced abortion
	636	Illegal induced abortion
	637	Unspecified abortion
	638	Attempted but failed abortion
	639	Complications following abortion and ectopic and molar pregnancies
Other complications of pregnancy, childbirth and the puerperium. Delivery without mention of complication (A120)	630	Hydatidiform mole
	631	Other abnormal product of conception
	633	Ectopic pregnancy
	643	Excessive vomiting in pregnancy
	644	Early or threatened labour
	645	Prolonged pregnancy
	646	Other complications of pregnancy, not elsewhere classified
	647	Infective and parasitic conditions in the mother classifiable elsewhere but complicating pregnancy, childbirth and the puerperium
	648	Other current conditions in the mother classifiable elsewhere but complicating pregnancy, childbirth and the puerperium
	650	Delivery in a completely normal case
	651	Multiple gestation
	652	Malposition and malpresentation of foetus
	653	Disproportion
	654	Abnormality of organs and soft tissues of pelvis
	655	Known or suspected foetal abnormality affecting management of mother
	656	Other foetal and placental problems affecting management of mother
	657	Polyhydramnios
	658	Other problems associated with amniotic cavity and membranes
	659	Other indications for care or intervention related to labour and delivery and not elsewhere classified
	660	Obstructed labour
	661	Abnormality of forces of labour
	662	Long labour
	663	Umbilical cord complications
	664	Trauma to perineum and vulva during delivery
	665	Other obstetrical trauma
	668	Complications of the administration of anaesthetic or other sedation in labour and delivery
	669	Other complications of labour and delivery, not elsewhere classified
	672	Pyrexia of unknown origin during the puerperium
	674	Other and unspecified complications of the puerperium, not elsewhere classified
	675	Infections of the breast and nipple associated with childbirth
	676	Other disorders of the breast associated with childbirth, and disorders of lactation
	677	Late effect of complication of pregnancy, childbirth and the puerperium

† Group names are based on the content of the ICD-7, list A (Intermediate list of 150 causes for tabulation of morbidity and mortality).
