# Supplementary material for: Women's Education Level, Maternal Health Facilities, Abortion Legislation and Maternal Deaths: A Natural Experiment in Chile from 1957 to 2007
Source: PLoS One. 2012 May 4;7(5):e36613. doi: 10.1371/journal.pone.0036613 (PMC3344918; doi:10.1371/journal.pone.0036613)
Supplement: Table S5 — International Classification of Diseases (ICD) 10th version for classifying maternal death causes in Chile. Homologation with five groups from ICD 7th version, list A. (PDF) [file pone.0036613.s011.pdf]

Table S5. International Classification of Diseases (ICD) 10th version for classifying maternal death causes in Chile. Homologation with five groups from ICD 7th version, list A.

Group List A ICD-7†	ICD-10 (1997-present)	Causes of death
Sepsis of pregnancy, childbirth and the puerperium (A115)	O22	Venous complications in pregnancy
	O23	Infections of genitourinary tract in pregnancy.
	O85	Puerperal sepsis
	O86	Other puerperal infections
	O87	Venous complications in the puerperium
	O88	Obstetric embolism
Toxaemias of pregnancy and the puerperium (A116)	O10	Pre-existing hypertension complicating pregnancy, childbirth and the puerperium
	O11	Pre-existing hypertensive disorder with superimposed proteinuria
	O12	Gestational [pregnancy-induced] oedema and proteinuria without hypertension
	O13	Gestational [pregnancy-induced] hypertension without significant proteinuria
	O14	Gestational [pregnancy-induced] hypertension with significant proteinuria
	O15	Eclampsia
	O16	Unspecified maternal hypertension
Haemorrhage of pregnancy and childbirth (A117)	O20	Haemorrhage in early pregnancy
	O43	Placental disorders
	O44	Placenta praevia
	O45	Premature separation of placenta [abruptio placentae]
	O46	Antepartum haemorrhage, not elsewhere classified
	O67	Labour and delivery complicated by intrapartum haemorrhage, not elsewhere classified
	O72	Postpartum haemorrhage
	O73	Retained placenta and membranes, without haemorrhage
Abortion with and without mention of sepsis or toxaemia (A118 and A119)	O03	Spontaneous abortion
	O04	Medical abortion
	O05	Other abortion
	O06	Unspecified abortion
	O07	Failed attempted abortion
	O08	Complications following abortion and ectopic and molar pregnancy
Other complications of pregnancy, childbirth and the puerperium. Delivery without mention of complication (A120)	O00	Ectopic pregnancy
	O01	Hydatidiform mole
	O02	Other abnormal products of conception
	O21	Excessive vomiting in pregnancy
	O24	Diabetes mellitus in pregnancy
	O25	Malnutrition in pregnancy
	O26	Maternal care for other conditions predominantly related to pregnancy
	O28	Abnormal findings on antenatal screening of mother
	O29	Complications of anaesthesia during pregnancy
	O30	Multiple gestation
	O31	Complications specific to multiple gestation
	O32	Maternal care for known or suspected malpresentation of fetus
	O33	Maternal care for known or suspected disproportion
	O34	Maternal care for known or suspected abnormality of pelvic organs
	O35	Maternal care for known or suspected fetal abnormality and damage
	O36	Maternal care for other known or suspected fetal problems
	O40	Polyhydramnios
	O41	Other disorders of amniotic fluid and membranes
	O42	Premature rupture of membranes
	O47	False labour
	O48	Prolonged pregnancy
	O60	Preterm labour and delivery
	O61	Failed induction of labour
	O62	Abnormalities of forces of labour
	O63	Long labour
	O64	Obstructed labour due to malposition and malpresentation of fetus
	O65	Obstructed labour due to maternal pelvic abnormality
	O66	Other obstructed labour
	O68	Labour and delivery complicated by fetal stress [distress]
	O69	Labour and delivery complicated by umbilical cord complications
	O70	Perineal laceration during delivery
	O71	Other obstetric trauma
	O74	Complications of anaesthesia during labour and delivery
	O75	Other complications of labour and delivery, not elsewhere classified

† Group names are based on the content of the ICD-7, list A (Intermediate list of 150 causes for tabulation of morbidity and mortality).

Table S5. International Classification of Diseases (ICD) 10th version for classifying maternal death causes in Chile. Homologation with five groups from ICD 7th version, list A.

(Table S5, continued)

Group List A ICD-7†	ICD-10 (1997-present)	Causes of death
Other complications of pregnancy, childbirth and the puerperium. Delivery without mention of complication (A120)	O80	Single spontaneous delivery
	O81	Single delivery by forceps and vacuum extractor
	O82	Single delivery by caesarean section
	O83	Other assisted single delivery
	O84	Multiple delivery
	O89	Complications of anaesthesia during the puerperium
	O90	Complications of the puerperium, not elsewhere classified
	O91	Infections of breast associated with childbirth
	O92	Other disorders of breast and lactation associated with childbirth
	O94	Sequelae of complication of pregnancy, childbirth and the puerperium
	O95	Obstetric death of unspecified cause
	O96	Death from any obstetric cause occurring more than 42 days but less than one year after delivery
	O97	Death from sequelae of direct obstetric causes
	O98	Maternal infectious and parasitic diseases classifiable elsewhere but complicating pregnancy, childbirth and the puerperium
	O99	Other maternal diseases classifiable elsewhere but complicating pregnancy, childbirth and the puerperium

† Group names are based on the content of the ICD-7, list A (Intermediate list of 150 causes for tabulation of morbidity and mortality).
