# Supplementary material for: Women's Education Level, Maternal Health Facilities, Abortion Legislation and Maternal Deaths: A Natural Experiment in Chile from 1957 to 2007
Source: PLoS One. 2012 May 4;7(5):e36613. doi: 10.1371/journal.pone.0036613 (PMC3344918; doi:10.1371/journal.pone.0036613)
Supplement: Table S6 — Time series of maternal deaths and selected indicators, Chile 1957–2007. (PDF) [file pone.0036613.s012.pdf]

Table S6. Time series of maternal deaths and selected indicators, Chile 1957 – 2007.

Year	Population at Fertile Age	Live Births	Maternal Deaths	MM Ratio†	MM Rate††
1957	1,646,472	271,905	736	270.7	44.7
1958	1,687,468	273,793	769	280.9	45.6
1959	1,728,465	276,686	672	242.9	38.9
1960	1,768,652	287,063	789	274.9	44.6
1961	1,815,443	296,230	870	293.7	47.9
1962	1,862,235	303,249	787	259.5	42.3
1963	1,909,027	306,575	733	239.1	38.4
1964	1,955,818	303,219	721	237.8	36.9
1965	1,967,861	302,401	787	260.3	40.0
1966	2,010,318	294,438	676	229.6	33.6
1967	2,052,776	273,399	630	230.4	30.7
1968	2,095,233	262,036	550	209.9	26.3
1969	2,137,691	252,157	454	180.1	21.2
1970	2,219,757	251,231	451	179.5	20.3
1971	2,259,149	261,021	335	128.3	14.8
1972	2,298,543	269,576	306	113.5	13.3
1973	2,337,936	291,277	331	113.6	14.2
1974	2,377,329	278,165	313	112.5	13.2
1975	2,548,645	266,513	306	114.8	12.0
1976	2,585,820	256,641	253	98.6	9.8
1977	2,622,995	242,847	214	88.1	8.2
1978	2,660,171	245,763	170	69.2	6.4
1979	2,697,346	251,745	155	61.6	5.8
1980	2,911,821	262,030	135	51.5	4.6
1981	2,959,928	273,882	116	42.4	3.9
1982	3,008,035	278,000	144	51.8	4.8
1983	3,056,142	259,888	107	41.2	3.5
1984	3,104,249	262,317	94	35.8	3.0
1985	3,240,601	263,039	132	50.2	4.1
1986	3,297,507	272,941	129	47.3	3.9
1987	3,354,413	279,367	135	48.3	4.0
1988	3,411,319	297,823	123	41.3	3.6
1989	3,468,226	306,080	125	40.8	3.6
1990	3,552,327	309,220	123	39.8	3.5
1991	3,605,504	300,740	106	35.3	2.9
1992	3,658,683	294,218	91	30.9	2.5
1993	3,711,862	289,419	100	34.6	2.7
1994	3,765,042	285,228	73	25.6	1.9
1995	3,818,221	275,760	86	31.2	2.3
1996	3,874,803	272,163	63	23.2	1.7
1997	3,931,387	265,493	61	23.0	1.6
1998	3,987,971	261,802	55	21.0	1.4
1999	4,044,553	254,096	60	23.6	1.5
2000	4,101,137	252,155	49	19.4	1.2
2001	4,156,091	248,651	45	18.1	1.1
2002	4,211,046	241,027	42	17.4	1.0
2003	4,265,999	236,223	30	12.7	0.7
2004	4,320,954	232,588	42	18.1	1.0
2005	4,375,908	232,092	48	20.7	1.1
2006	4,411,544	233,104	47	20.2	1.1
2007	4,447,180	242,054	44	18.2	1.0

† Referred to maternal mortality ratio, number of deaths per 100,000 live births

† Referred to maternal mortality rate, number of deaths per 100,000 women of fertile age
