# Supplementary material for: Women's Education Level, Maternal Health Facilities, Abortion Legislation and Maternal Deaths: A Natural Experiment in Chile from 1957 to 2007
Source: PLoS One. 2012 May 4;7(5):e36613. doi: 10.1371/journal.pone.0036613 (PMC3344918; doi:10.1371/journal.pone.0036613)
Supplement: Table S7 — Direct comparison of the maternal mortality ratio (MMR) estimates by the World Health Organization (WHO) report with available official domestic data in eight countries of the American continent in 2008. These countries are classified in list A according to the completeness of their vital statistics by United Nations (i.e. civil registration of deaths is virtually complete). An important overestimation (e.g. 35.4% for Colombia, 48.6% for Mexico, 57.6% for Chile and 76.3% for Argentina) can be observed in WHO maternal mortality estimates for these countries with full official records of maternal deaths. In consequence, progress in maternal health seems to be underestimated in several Latin American countries by WHO technical reports. (PDF) [file pone.0036613.s013.pdf]

Table S7. Direct comparison of the maternal mortality ratio (MMR) † estimates by the World Health Organization (WHO) report[a] with available official domestic data in eight countries of the American continent in 2008. These countries are classified in list A according to the completeness of their vital statistics by United Nations (*i.e.* civil registration of deaths is virtually complete). An important overestimation (*e.g.* 35.4% for Colombia, 48.6% for Mexico, 57.6% for Chile and 76.3% for Argentina) can be observed in WHO maternal mortality estimates for these countries with full official records of maternal deaths. In consequence, progress in maternal health seems to be underestimated in several Latin American countries by WHO technical reports.

Country	Live births	Maternal deaths	MMR directly calculated†	MMR by WHO report	Difference††	Overestimation (%)
Canada [b]	377,886	34	9.0	12	3	33.3
Chile [c]	248,366	41	16.5	26	9.5	57.6
United States [d, e]	4,247,694	795	18.7	24	5.3	28.3
Costa Rica [f]	75,187	25	33.3	44	10.7	32.1
Cuba [g]	122,569	57	46.5	53	6.5	14
Argentina [h]	746,460	296	39.7	70	30.3	76.3
Mexico [i,j]	1,955,284	1,119	57.2	85	27.8	48.6
Colombia [k, l]	715,453	449	62.8	85	22.2	35.4

MMR refers to the maternal mortality ratio per 100,000 live births. †MMR = (Maternal deaths / Live births) x 100,000. [a]World Health Organization (2010) Trends in maternal mortality: 1990 to 2008. †† Calculated as the difference between the MMR directly calculated and the MMR estimated by WHO[a]. Official domestic data were obtained from: [b]Statistics Canada (STATCAN), [c]Instituto Nacional de Estadística (INE) (2010) "Estadísticas Vitales, Informe Anual 2008", [d]Martin, J. *et al.* (2010) "National Vital Statistics Reports. Births: Final Data for 2008", [e]Miniño, A. *et al.* (2011) "National Vital Statistics Reports. Deaths: Final Data for 2008", [f]Instituto Nacional de Estadística y Censo (INEC) "Estadísticas vitales 2008", [g]Oficina Nacional de Estadísticas (ONE) "Anuario Estadistico de Cuba 2010. Maternal mortality rate and its causes", [h]Dirección de Estadísticas e Información de Salud (DEIS) "Estadisticas Vitales. Información Básica 2008", [i]Instituto Nacional de Estadística y Geografía (INEGI) "Consulta interactiva de datos. Estadísticas de natalidad", [j]Sistema Nacional de Información en Salud (SINAIS) "Razón de mortalidad materna y defunciones por año de registro, 2002-2008", [k]Departamento Administrativo Nacional de Estadística (DANE) "Estadísticas vitales. Defunciones no fetales año 2008. Cuadro 8" and [l]Departamento Administrativo Nacional de Estadística (DANE) "Estadísticas vitales. Nacimientos año 2008. Cuadro 1".
