# Supplementary material for: Women's Education Level, Maternal Health Facilities, Abortion Legislation and Maternal Deaths: A Natural Experiment in Chile from 1957 to 2007
Source: PLoS One. 2012 May 4;7(5):e36613. doi: 10.1371/journal.pone.0036613 (PMC3344918; doi:10.1371/journal.pone.0036613)
Supplement: Table S8 — Abortion hospitalizations in Chile based on official data and estimated proportions for clinical spontaneous abortions and clandestine induced abortions for the period 2001–2008. During this period, hospitalizations from clandestine abortion complications are estimated between 12% and 19% out of total hospitalizations by abortion (calculated as the difference between expected hospitalizations by clinical spontaneous abortion and observed hospitalizations by abortion; for additional information, see Appendix S2). (PDF) [file pone.0036613.s014.pdf]

Table S8. Abortion hospitalizations in Chile based on official data and estimated proportions for clinical spontaneous abortions and clandestine induced abortions for the period 2001-2008. During this period, hospitalizations from clandestine abortion complications are estimated between 12% and 19% out of total hospitalizations by abortion (calculated as the difference between expected hospitalizations by clinical spontaneous abortion and observed hospitalizations by abortion; for additional information, see Appendix S2).

Year	Observed Live Births†	Observed Abortion hospitalizations [a]	Ratio	Expected clinical spontaneous abortions [b]	Excess in abortion hospitalizations‡	Percent (%) [c]
2001	248,651	34,479	7,1	29,238	5,241	15,2
2002	241,027	34,968	6,8	28,380	6,588	18,8
2003	236,223	33,490	7,0	27,856	5,634	16,8
2004	232,588	33,835	6,8	27,365	6,470	19,1
2005	232,092	33,184	7,0	27,422	5,762	17,4
2006	233,104	33,145	7,0	27,488	5,657	17,1
2007	242,054	32,532	7,4	28,579	3,953	12,2
2008	246,581	33,428	7,4	29,293	4,135	12,4

†Based on data of observed live births (uncorrected) by the Chilean National Institute of Statistics, INE (2010) "Estadísticas Vitales, Informe Anual 2008". [a] Official number published by the Ministry of Health. Ratio refers to live births/abortions hospitalizations ratio. [b] Estimated applying the probabilities of the study by Wang *et al*. (2003) "Conception, early pregnancy loss, and time to clinical pregnancy: a population-based prospective study." Fertil Steril 79(3): 577. ‡Estimated as the difference between observed and expected hospitalizations from abortion. [c] Estimated proportion of hospitalizations possibly related to induced abortions.
